# R-loops and D-loops: a delicate balance in genomic stability and instability

**DOI:** 10.1186/s12964-026-03033-5

**Published:** 2026-07-03

**Authors:** Zhendong Qin, Mingjun Lu, Jiaqi Zhao, Jiabao Hou, Jingwei Guo, Jinghong Wu, Chenyang Wang, Xiaoyue Zhu, Teng Ma

**Affiliations:** Cancer Research Center, Beijing Chest Hospital, Capital Medical University, Beijing Tuberculosis and Thoracic Tumor Research Institute, Beijing, 101149 China

**Keywords:** R-loop, D-loop, Genome stability, DNA replication, Transcription, Homologous recombination

## Abstract

R-loops and D-loops are three-stranded nucleic acid structures that have emerged as central regulators of genome stability, gene expression, and DNA metabolism. R-loops form co-transcriptionally or post-transcriptionally when nascent RNA re-anneals with the template DNA strand, generating an RNA: DNA hybrid that displaces the non-template strand into a single-stranded state. These structures are enriched at CpG island promoters, transcription termination sites, and immunoglobulin class-switch regions, where they coordinate transcription regulation, chromatin remodeling, and DNA damage signaling. D-loops are formed when a single-stranded DNA segment pairs with one strand of a duplex and displaces the other, arising through context-dependent mechanisms that include RAD51- or DMC1-mediated strand invasion in homologous recombination, shelterin-assisted invasion at telomeres, and replication-coupled strand displacement at the mitochondrial DNA origin. They serve as indispensable intermediates in double-strand break repair, telomere maintenance, and mitochondrial DNA replication. Recent cryo-electron microscopy studies have resolved the stepwise RAD51-mediated strand exchange mechanism at near-atomic resolution, substantially advancing structural understanding of D-loop biogenesis. Despite their differences in molecular composition, both structures remodel Watson-Crick base pairing and, when dysregulated, are associated with replication fork stalling, transcription-replication conflicts, and aberrant recombination. This review systematically compares the structural features, formation mechanisms, regulatory networks, and biological functions of R-loops and D-loops, with emphasis on their convergent roles in safeguarding genome integrity. We further discuss rapidly evolving detection technologies and emerging therapeutic strategies targeting these structures in cancer and neurodegeneration, identifying key unresolved questions for future investigation.

## Introduction

Since the double helix structure of DNA was proposed by Watson and Crick in 1953, it has been regarded as the canonical model for genetic information storage [1]. However, researchers have discovered that various non-canonical nucleic acid structures are prevalent across genomes. Among them, three-stranded nucleic acid structures serve as crucial regulators of genomic architecture and function, playing an indispensable role in genomic regulation.

R-loops and D-loops are two important three-stranded nucleic acid structures. While they share similar topological features, they differ fundamentally in molecular composition and formation mechanisms. R-loops, first visualized by Thomas et al. using electron microscopy [2], arise when nascent transcripts hybridize with template DNA. During transcription, RNA can form persistent three-stranded structures with DNA, where an RNA-DNA hybrid displaces the original DNA duplex. Genome-wide DRIP-seq (DNA: RNA immunoprecipitation sequencing) analyses have demonstrated that R-loops are widely distributed in mammalian cells, particularly enriched at CpG island promoters, transcription termination sites, and immunoglobulin class-switch regions [3–5]. Advances in detection technologies, from immunoprecipitation-based approaches to antibody-free methods achieving base-pair resolution, have substantially refined our understanding of R-loop distribution and are discussed in detail in the R-loop Detection Methods section.

D-loops, initially observed in mitochondrial DNA replication by Kasamatsu and Vinograd [6, 7], form through strand invasion processes. Specifically, D-loops are intermediate structures formed during homologous recombination, where a single-stranded DNA invades a homologous DNA duplex and displaces one of its strands. The characterization of D-loop formation during RecA-mediated homologous recombination was elucidated in subsequent studies throughout the late 1970s and early 1980s [8–10]. The mechanistic understanding of D-loop formation has advanced considerably since the first crystal structures of the bacterial recombinase RecA (*Escherichia coli*) bound to DNA substrates [11]. Recent cryo-electron microscopy studies have resolved the stepwise mechanism of RAD51-mediated strand exchange at near-atomic resolution [12–15]. Similarly, advanced detection methods including electron microscopy, chromatin immunoprecipitation (ChIP), and high-throughput sequencing techniques like ChIP-seq enable us to map the genome-wide distribution of D-loops with increasing precision [16, 17].

The metabolism of R-loops and D-loops is evolutionarily conserved across the three domains of life. R-loops have been documented from bacteria to mammals, where they regulate transcription, DNA replication, and genome stability [5, 18]. They were first characterized in *Escherichia coli* as RNase HI-processed primers for ColE1 plasmid replication [19], and subsequent genome-wide profiling has identified them as pervasive chromatin features in *Saccharomyces cerevisiae*, *Arabidopsis thaliana*, and mammalian cells [20]. The R-loop resolution machinery is similarly conserved, exemplified by the structural and functional homology between yeast Sen1 and its human orthologue SETX [21]. D-loop biology shows parallel conservation: the recombinase superfamily that catalyzes D-loop formation, including bacterial RecA, archaeal RadA, and eukaryotic RAD51 and DMC1, descends from a common ancestor predating the divergence of the three domains and assembles strikingly similar nucleoprotein filaments [22]. This deep conservation indicates that R-loop and D-loop metabolism represents an ancient and indispensable feature of cellular life, providing a framework for translating mechanistic insights from model organisms to human disease.

**Fig. 1:**
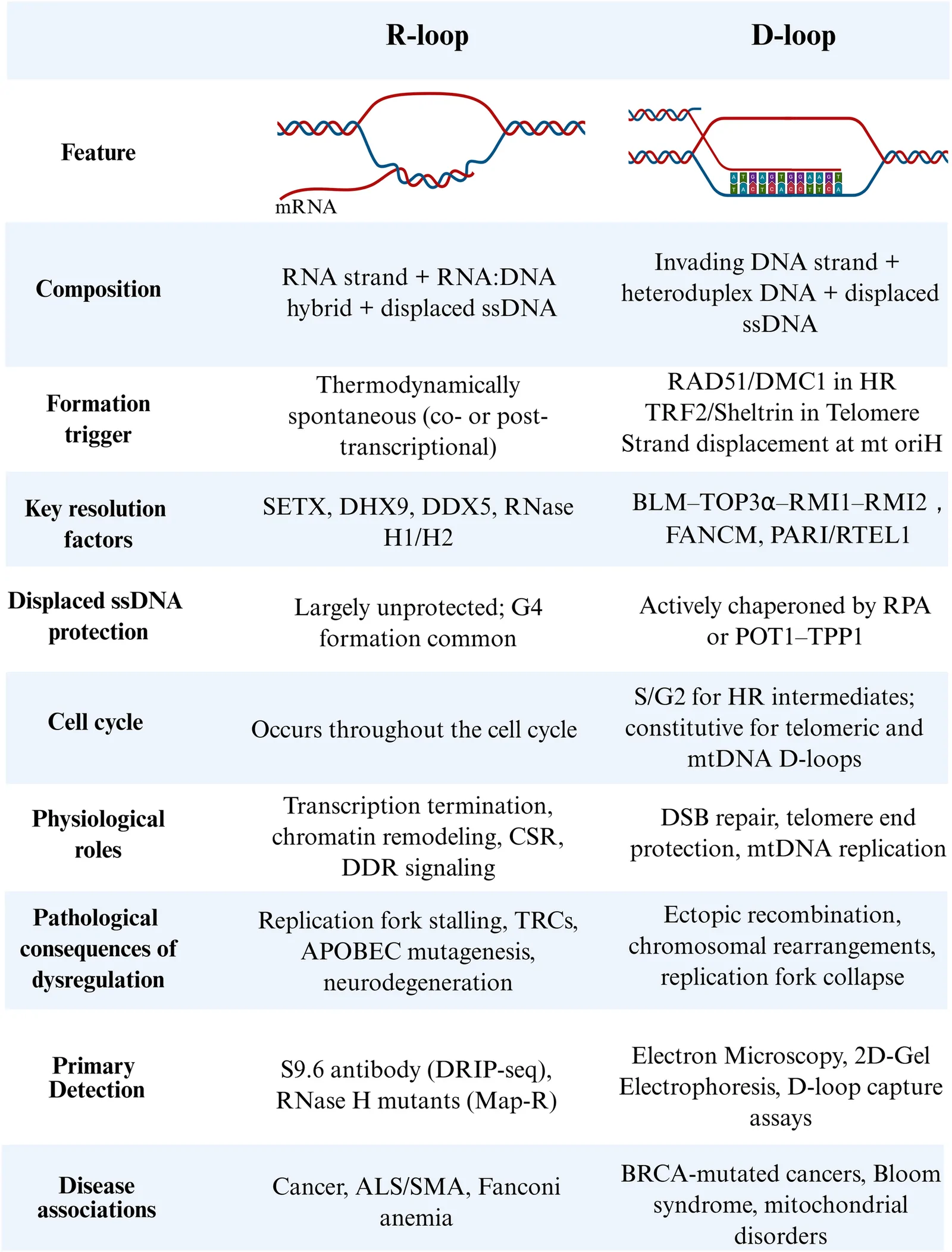
Comparative overview of R-loops and D-loops. Side-by-side comparison of the structural, mechanistic, regulatory, and pathological features of R-loops and D-loops. R-loops are three-stranded nucleic acid structures in which a nascent mRNA (red) anneals to the template DNA, exposing the non-template strand as displaced ssDNA [23]; their formation is thermodynamically driven and requires no enzymatic catalyst. D-loops are homologous-recombination intermediates in which a RAD51/DMC1-coated invading ssDNA (red, with Watson–Crick base pairing to the homologous template indicated by colored rungs) displaces one strand of the recipient duplex as a single-stranded loop [24, 25]. Beyond canonical HR, constitutive D-loop-like structures persist at telomeres (T-loops) and within the mitochondrial non-coding region (mtDNA D-loop) [26]. Resolution factors, displaced-ssDNA chaperones, primary detection methods, and representative disease associations are summarized in the corresponding rows. Abbreviations: ssDNA, single-stranded DNA; HR, homologous recombination; DSB, double-strand break; CSR, class-switch recombination; DDR, DNA damage response; TRC, transcription–replication conflict; G4, G-quadruplex; mtDNA, mitochondrial DNA; APOBEC, apolipoprotein B mRNA-editing enzyme catalytic polypeptide-like; DRIP-seq, DNA: RNA hybrid immunoprecipitation sequencing; Map-R, mapping of R-loops; 2D-Gel, two-dimensional gel electrophoresis; ALS, amyotrophic lateral sclerosis; SMA, spinal muscular atrophy

Although the biological functions of R-loops and D-loops have been extensively studied in isolation, their comparative analysis remains fragmented. Given their structural similarities, their roles in genome stability, and their potential regulatory mechanisms in disease development, this review aims to provide a comprehensive comparative analysis of R-loops and D-loops, thereby providing a framework to understand their distinct and overlapping contributions to genome biology and disease (Fig. 1).

## R-loop

### Basic structure

R-loops are three-stranded nucleic acid structures in which single-stranded RNA forms an RNA: DNA hybrid duplex with one strand of double-stranded DNA (typically the template strand) through Watson-Crick base pairing, while simultaneously displacing the other DNA strand (non-template strand) into a single-stranded state [5, 27]. R-loops are highly conserved three-stranded nucleic acid structures prevalent across diverse biological species that perform essential functions in cellular physiology [28, 29]. A comprehensive elucidation of their unique structural characteristics is of fundamental importance for fully understanding their multifaceted functions in gene expression, the maintenance of genomic stability, and the pathogenesis of human diseases.

From the perspective of structural stability, R-loops exhibit remarkable thermodynamic stability characteristics. Recent thermodynamic and nuclear magnetic resonance analyses have revealed that RNA: DNA hybrid duplexes adopt an intermediate conformation between canonical A-form and B-form helices [30, 31]. In vitro biochemical studies demonstrate that the formation of stable R-loops typically requires an RNA: DNA hybrid region of at least approximately 100 base pairs in length [32]. The exceptional stability of RNA: DNA hybrids primarily derives from their unique structural features.

Molecular dynamics simulations and structural analyses have established that although RNA: DNA hybrids can adopt conformations ranging from A-like to B-form depending on sequence composition, they generally favor an A-form-like geometry [33, 34]. This A-form-like conformation is characterized by a shorter helical pitch (approximately 2.8 nm per turn), a narrower and deeper major groove, and enhanced base stacking interactions compared to the classical B-form DNA double helix [35, 36]. The resulting lower free energy state contributes to the exceptional thermodynamic stability of RNA: DNA hybrids [37].

A 2024 study employing coarse-grained modeling of DNA-RNA hybrids (oxNA model) has further elucidated these structural properties [38]. This oxNA model revealed that DNA: RNA hybrids naturally adopt an intermediate helix geometry between A-form and B-form without explicit structural constraints, with this compromise affecting their thermodynamic properties and biological functions. The model demonstrates that the short range of hydrogen-bonding interactions forces base pairs to lie approximately in the same plane, resulting in the observed structural intermediate [38]. Complementary molecular dynamics studies on DNA and RNA double helices formed by trinucleotide repeats and associated DNA-RNA hybrids have provided additional insights into sequence-dependent structural variations [39].

The transition from B-form to A-like hybrid duplexes is guided by purine-pyrimidine asymmetry and significantly influences the hydration number and stability of these structures under cellular crowding conditions [30]. Recent investigations into in-cell stability of RNA/DNA hybrid duplexes have revealed temperature-dependent characteristics that affect their therapeutic applications [30]. Consequently, once formed, R-loops generally do not dissociate spontaneously and require active resolution by dedicated RNA: DNA helicases (such as SETX, DDX5, and DHX9) or ribonucleases (such as RNase H1/H2) [40, 41].

### Regulation of formation and stability

#### Origin and formation mechanisms

Two mechanistic models have been proposed to explain how R-loops originate during transcription. The “post-transcriptional” model, also referred to as the “thread-back” mechanism, posits that nascent RNA, once released from the elongation complex, re-anneals with the template DNA strand in a retrograde fashion [42]. The alternative “co-transcriptional” model proposes that RNA remains continuously base-paired with the template strand during synthesis, extending the hybrid as transcription proceeds [43]. Although both models are conceptually plausible and are not mutually exclusive, current experimental evidence, including direct single-molecule visualization of transcription–replication conflicts and post-replicative RNA: DNA hybrids, predominantly supports the thread-back (post-transcriptional) route [44, 45]. Nevertheless, neither model alone fully explains the cell-cycle specificity or locus-selectivity of R-loop formation observed in vivo, suggesting that additional layers of regulation modulate which mechanism predominates at a given genomic context. This mechanistic ambiguity represents an important unresolved question in the field.

**Fig. 2:**
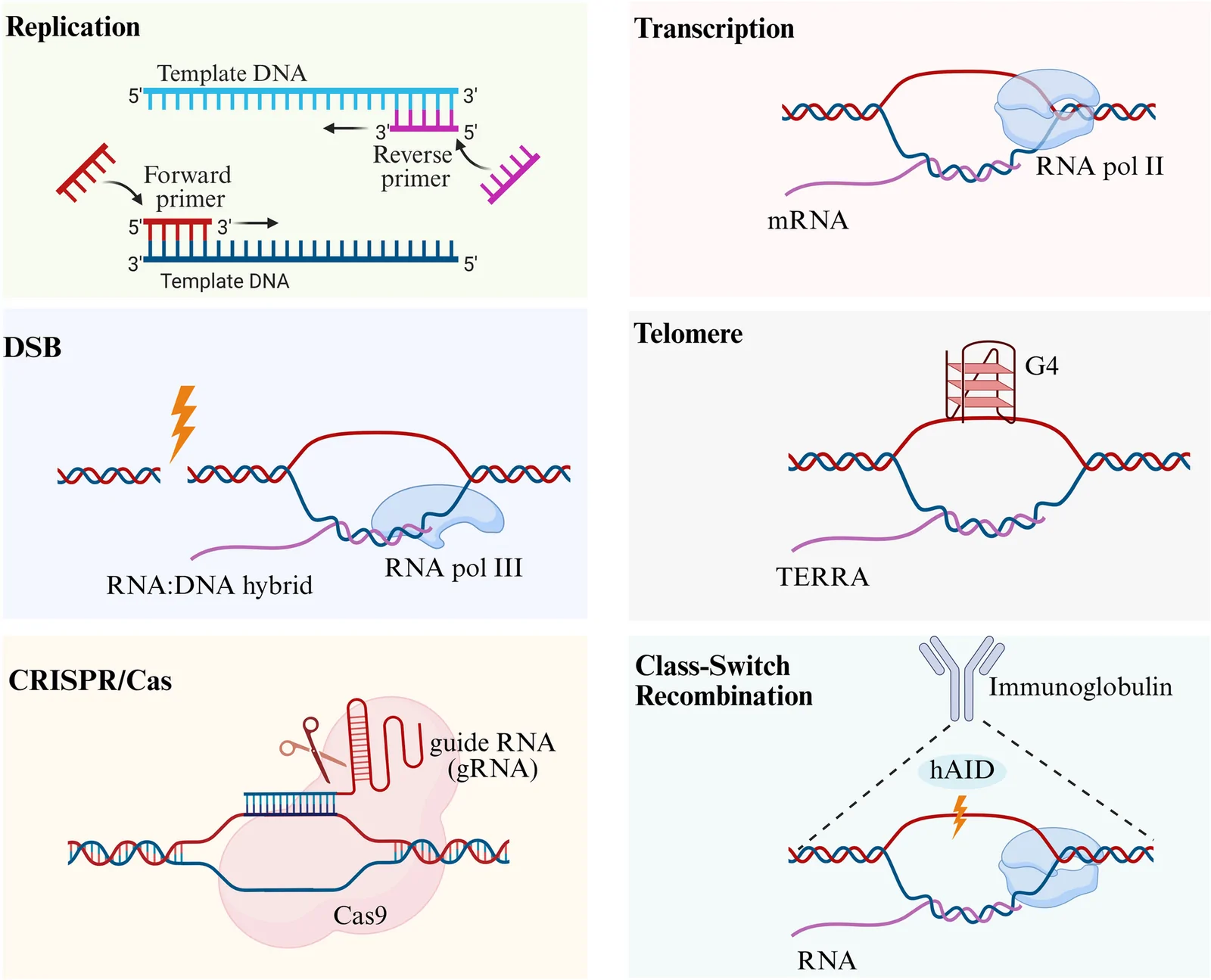
Biological contexts of RNA:DNA hybrid formation. Schematic illustration of six distinct biological scenarios in which RNA: DNA hybrids arise. During DNA replication, RNA primers synthesized by primase serve as initiation points for the leading and lagging strands [57]. At double-strand breaks (DSBs), RNA polymerase III-transcribed RNA can hybridize at damage sites to facilitate repair [48]. CRISPR/Cas9-mediated cleavage depends on guide RNA forming a transient RNA: DNA hybrid (R-loop) with the target strand [55]. During transcription elongation, RNA polymerase II promotes co-transcriptional R-loop formation, particularly at telomeres through TERRA transcripts [58]. Telomeric R-loops are further stabilized by the formation of G-quadruplex (G4) structures in the displaced non-template strand [59]. In immunoglobulin class-switch recombination (CSR), R-loops formed at switch regions recruit activation-induced cytidine deaminase (hAID), initiating downstream recombination [60]

Beyond the canonical transcription-coupled pathway, R-loops can also arise from a variety of non-transcriptional and context-specific mechanisms. During DNA replication, RNA primers synthesized by primase serve as the initial nucleating scaffold for R-loop-like RNA: DNA hybrid structures at both the leading and lagging strands; these post-replicative hybrids, which form after replication fork passage through transcribed regions, represent a mechanistically distinct source of R-loops [46, 47]. In the context of DNA double-strand breaks (DSBs), RNA Polymerase III (RNA Pol III) is actively recruited to DSB sites by the MRN complex and synthesizes RNA strands that hybridize with the resected 3′ssDNA overhangs to form RNA: DNA hybrids, thereby implicating R-loop formation as an integral component of the DNA damage response [48, 49]. At telomeres, the long non-coding RNA TERRA (Telomeric Repeat-containing RNA), transcribed by RNA Pol II from subtelomeric promoters, can invade the telomeric duplex to form R-loops, which are further stabilized by the propensity of G-rich telomeric sequences to adopt G-quadruplex (G4) secondary structures on the displaced single-stranded DNA strand [50–52]. During class switch recombination (CSR) in B lymphocytes, R-loops formed at switch regions expose single-stranded DNA as substrates for activation-induced cytidine deaminase (hAID), coupling R-loop biology directly to immunoglobulin diversification [4, 53]. In the context of CRISPR/Cas genome editing, the Cas9–sgRNA complex displaces the non-template DNA strand upon PAM recognition and progressive target invasion, generating a defined R-loop intermediate that is mechanistically essential for Cas9-mediated DNA cleavage [54, 55]. Furthermore, small non-coding RNAs and lncRNAs can invade duplex DNA to form R-loop-like structures in trans [56]. The relative quantitative contribution of these alternative pathways to the total cellular R-loop burden has not been systematically established, constituting a significant gap in our understanding of R-loop biogenesis (Fig. 2).

Recent work has demonstrated that R-loops are not merely passive by-products of transcription but can actively regulate gene regulatory programs. R-loops have been shown to coordinate RNA polymerase II (RNA Pol II) transcriptional reprogramming at developmentally critical transitions. For instance, during the maternal-to-zygotic transition, CG-poor R-loops inhibit DDX21 helicase activity, restrict CDK9 release, and enforce RNA Pol II pausing for precise temporal control of zygotic genome activation [61]. These findings reframe R-loops as context-dependent regulatory signals rather than simply threats to genome integrity, and underscore the importance of distinguishing “physiological” from “pathological” R-loop states, a distinction that current detection technologies do not yet resolve with sufficient resolution.

#### Intrinsic sequence and structural determinants

R-loop formation is strongly influenced by intrinsic features of the underlying DNA sequence. Guanine (G)-rich sequences on the non-template strand represent hotspots for R-loop formation genome-wide, attributable primarily to the enhanced thermodynamic stability of G·C-rich RNA: DNA hybrids [30, 37, 62]. DRIP-seq and its derivatives consistently identify G-richness as one of the strongest predictors of R-loop occupancy [62, 63]. However, G-richness alone is not sufficient; flanking sequence context, GC skew, and transcription rate all modulate the propensity of a given locus to form persistent R-loops. This highlights the limitations of sequence-based predictions alone and underscores the need for experimental validation.

The displaced single-stranded non-template DNA within an R-loop frequently adopts G-quadruplex structures when G-rich, creating a mutually reinforcing G-loop architecture [59, 64]. G4 stabilization inhibits re-annealing of the displaced strand with the template, thereby prolonging R-loop lifetime [65]. Conversely, RNA transcripts can actively regulate G4 landscapes through coordinated G-loop assembly and disassembly in a process involving ATM/ATR kinases, BRCA2, and RAD51-mediated RNA invasion [66]. The bidirectionality of this G4–R-loop relationship, wherein each structure can promote the formation and stabilization of the other, creates a positive feedback loop with amplifying consequences for transcriptional output and genome stability [67]. The extent to which this feedback operates in a physiological versus pathological register likely depends on cellular context and the availability of resolving factors, but the mechanistic determinants of this switch remain poorly defined.

DNA topology constitutes another critical intrinsic determinant. Negative superhelical tension generated behind elongating RNA polymerase lowers the energetic barrier for RNA: template DNA hybridization by facilitating local strand separation [68]. DNA topoisomerase I (TOP1) normally dissipates this negative supercoiling; cells lacking TOP1 accumulate excessive negative supercoiling, creating a permissive environment for R-loop formation, particularly at highly transcribed loci such as ribosomal RNA genes [69]. While the role of negative supercoiling is well established, the precise quantitative relationship between supercoiling density, transcription rate, and R-loop probability across different genomic contexts has not been rigorously modelled. This represents a gap that could, in principle, be addressed through integrated topological and single-molecule approaches.

#### Trans-acting regulatory factors: helicases and nucleases

R-loop homeostasis is actively maintained by a battery of trans-acting proteins that either disassemble the hybrid directly or degrade its RNA component. Among RNA: DNA helicases, Senataxin (SETX/Sen1), DHX9, AQR, and DDX5 are the best characterised resolving factors [40, 41]. SETX acts at transcription termination zones and DNA double-strand break (DSB) hotspots, and its functional cooperation with BRCA1 directly links R-loop homeostasis to homologous recombination (HR) repair [70]. RNase H1 and H2 resolve R-loops through hydrolytic degradation of the RNA strand within the hybrid [3]. While the functional roles of individual factors are increasingly understood, the hierarchical organisation of this resolving machinery remains incompletely characterised. Key unresolved questions include which factors act constitutively versus conditionally, and how they are recruited to specific loci.

A key conceptual advance in recent years is the recognition that several helicases serve dual and context-dependent roles: DHX9, for example, can function as both an R-loop inducer and a resolver depending on transcriptional context and binding partners [40]. Similarly, DDX21, a nucleolar DEAD-box helicase, couples RNA m6A deposition with transcription termination to prevent R-loop accumulation at highly transcribed loci [71]. This functional duality challenges the traditional binary classification of R-loop regulators as simply “promoters” or “suppressors” and implies that the net effect of any single factor on R-loop levels depends critically on its interaction partners and cellular state. This complexity poses a challenge for interpreting loss-of-function studies, which may conflate direct effects on R-loop formation with indirect effects on transcription, RNA processing, or repair.

The transcription-coupled nucleotide excision repair (TC-NER) pathway intersects with R-loop metabolism through the activity of CSB, XPG, and XPF–ERCC1, which participate in R-loop resolution alongside their canonical DNA repair functions [72, 73]. The Fanconi anaemia (FA) pathway, including FANCD2, FANCA, and FANCM, also contributes to suppressing R-loop-dependent replication stress. Cells deficient in FA factors accumulate R-loops and R-loop-dependent DNA breaks, and this accumulation can be suppressed by RNase H1 overexpression or transcription inhibition [74, 75]. However, the precise molecular mechanisms by which FA proteins promote R-loop removal remain to be fully elucidated. Key questions include whether they act through direct unwinding, facilitation of RNA degradation, or replication fork stabilisation [76]. The functional overlap between TC-NER and FA pathways in R-loop resolution suggests a degree of pathway redundancy that complicates genetic dissection of their individual contributions.

#### Chromatin architecture and epigenetic regulation

Chromatin structure and histone modification state critically modulate R-loop accessibility and stability. Trimethylation of histone H3 at lysine 9 (H3K9me3), a mark associated with constitutive heterochromatin, is generally associated with suppression of R-loop formation, likely by restricting RNA polymerase access and maintaining a compacted chromatin state [77]. Conversely, H3K27 acetylation and the resultant chromatin relaxation at active enhancers and promoters create a permissive environment for RNA: DNA hybrid formation [78]. Acute global depletion of R-loops in mouse embryonic stem cells leads to reduced H2A.Z occupancy and increased nucleosome density at active and bivalent promoters, revealing a reciprocal relationship whereby R-loops actively contribute to maintaining an open chromatin architecture [78]. This bidirectional coupling between R-loops and chromatin state introduces the possibility of self-reinforcing regulatory circuits. In such a circuit, R-loop formation promotes chromatin accessibility, which in turn facilitates further R-loop formation. However, the stability conditions and limits of such circuits remain poorly understood.

Linker histone H1, by contrast, suppresses R-loop formation in heterochromatic regions; H1 depletion leads to R-loop accumulation and genome instability, implicating chromatin compaction as an active barrier to R-loop propagation [77]. Together, these observations indicate that the chromatin landscape provides a spatial template that restricts R-loop formation to transcriptionally active euchromatic regions under normal conditions. A key open question is how the cell prevents ectopic R-loop formation in regions of transiently decompacted heterochromatin, for example during replication or following DNA damage. It also remains to be determined whether failures in this containment contribute to pathological R-loop accumulation in disease contexts.

#### Epitranscriptomic regulation: RNA modifications

Epitranscriptomic modifications, particularly N6-methyladenosine (m6A) and 5-methylcytosine (m5C), have emerged as key modulators of R-loop metabolism. METTL3-mediated m6A modification promotes R-loop formation near transcription termination sites, and METTL3 depletion markedly reduces R-loop levels [79]. In human pluripotent stem cells, m6A marks at RNA: DNA hybrids are recognised by distinct reader proteins with opposing functional outcomes: YTHDC1 promotes R-loop accumulation and recruits HR repair factors, while YTHDF2 facilitates degradation of m6A-marked RNA to promote R-loop resolution, and IGF2BPs stabilise R-loops through m6A-modified RNA binding [79, 80]. This “reader code” for m6A-marked R-loops represents a sophisticated regulatory logic analogous to histone modification readout, but several important questions remain open: How is the relative activity of competing readers determined at individual R-loop loci? Does m6A modification of the RNA component alter the thermodynamic stability of the hybrid itself, or does its regulatory effect operate exclusively through reader-mediated pathways? Addressing these questions will require methods capable of simultaneously profiling RNA modification and R-loop formation at single-molecule resolution.

m5C modification, deposited by TRDMT1, influences R-loop-mediated DNA damage repair pathway choice, specifically modulating the balance between HR and non-homologous end joining (NHEJ) [81]. The mechanistic basis for this pathway bias is not fully resolved, but likely involves changes in R-loop stability or downstream signalling at damage sites. Additionally, phosphorylated hTERT has been identified as a regulator of R-loop integrity through direct interaction with the FANC/BRCA protein network, extending the functional crosstalk between R-loop regulatory mechanisms and DNA damage repair beyond epitranscriptomic control [82]. The existence of multiple, partially overlapping epitranscriptomic regulatory inputs raises the question of whether these pathways act hierarchically or in a combinatorial fashion. This question is not well addressed by current studies, which are largely performed in single-perturbation systems.

#### Non-coding RNA contributions to R-loop regulation

Various non-coding RNAs (ncRNAs) participate in the regulation of R-loop dynamics, adding a further layer of regulatory complexity. The lncRNA TUG1 suppresses R-loop formation at specific microsatellite loci through interaction with DHX9, and loss of TUG1 leads to R-loop accumulation and impaired cancer cell proliferation [83]. CircRNAs can form circR-loop structures with their parental genomic loci, modulating transcriptional pausing, chromatin remodelling, and DNA damage responses [84, 85]. The p53-inducible circRNA circASCC3 promotes R-loop resolution by stabilizing DDX5 protein, thereby maintaining genomic stability and conferring chemotherapy resistance [86].

While these examples demonstrate that ncRNAs can both promote and suppress R-loop formation through distinct mechanisms, a systems-level understanding of ncRNA-mediated R-loop regulation is lacking. Most studies have investigated individual ncRNA–R-loop interactions in isolation; whether these interactions are coordinately regulated, whether they compete for shared protein intermediaries, and how their combined activity shapes the global R-loop landscape remain unresolved. Furthermore, the extent to which ncRNA-mediated R-loop regulation is context-specific has not been systematically established. It remains unclear whether this regulation is confined to particular cell types, developmental stages, or stress conditions.

#### RNA secondary structure and competitive inhibition of R-loop formation

The propensity of a nascent RNA to form intramolecular secondary structures (e.g., hairpins, stem-loops) competes directly with its capacity to engage in intermolecular hybridisation with the template DNA strand [87, 88]. When a transcript rapidly folds into stable secondary structures co-transcriptionally, the accessible single-stranded regions available for R-loop formation are reduced, thereby suppressing hybrid formation. This competition creates a kinetic gating mechanism by which RNA folding speed and thermodynamic stability effectively limit R-loop formation to transcripts that are either structurally disordered or fold too slowly to outcompete template re-annealing. RNA-binding proteins (RBPs) that stabilise nascent RNA in an unfolded conformation, including members of the SR protein family and the THO complex, thus act as indirect facilitators of R-loop prevention [89].

The quantitative relationship between RNA folding kinetics and R-loop probability has not been modelled rigorously in vivo, and whether this competition is a major determinant of cell-type- or locus-specific R-loop landscapes is unknown. Recent advances in co-transcriptional RNA structure probing (e.g., SHAPE-seq in cells) may provide the tools to address this question, but their integration with R-loop mapping approaches remains technically challenging.

#### Synthesis: a multi-layered regulatory network and key knowledge gaps

Taken together, R-loop formation and stability are governed by a multi-layered regulatory network in which sequence features, DNA topology, RNA modifications, RNA secondary structure, ncRNAs, chromatin state, and a repertoire of resolving enzymes all converge to determine the spatiotemporal R-loop landscape. Several integrative principles emerge from this body of evidence. First, R-loop formation is not simply a consequence of transcription but is actively shaped by the cellular environment; the same genomic locus may sustain persistent R-loops in one cell type or cell cycle phase while remaining largely free of R-loops in another. Second, the regulatory factors involved frequently exhibit functional duality, promoting or suppressing R-loops depending on context. Third, multiple regulatory layers (e.g., m6A readout, helicase activity, chromatin compaction) converge on common R-loop substrates, suggesting a high degree of regulatory redundancy that may buffer against perturbations but also poses challenges for therapeutic targeting.

**Fig. 3:**
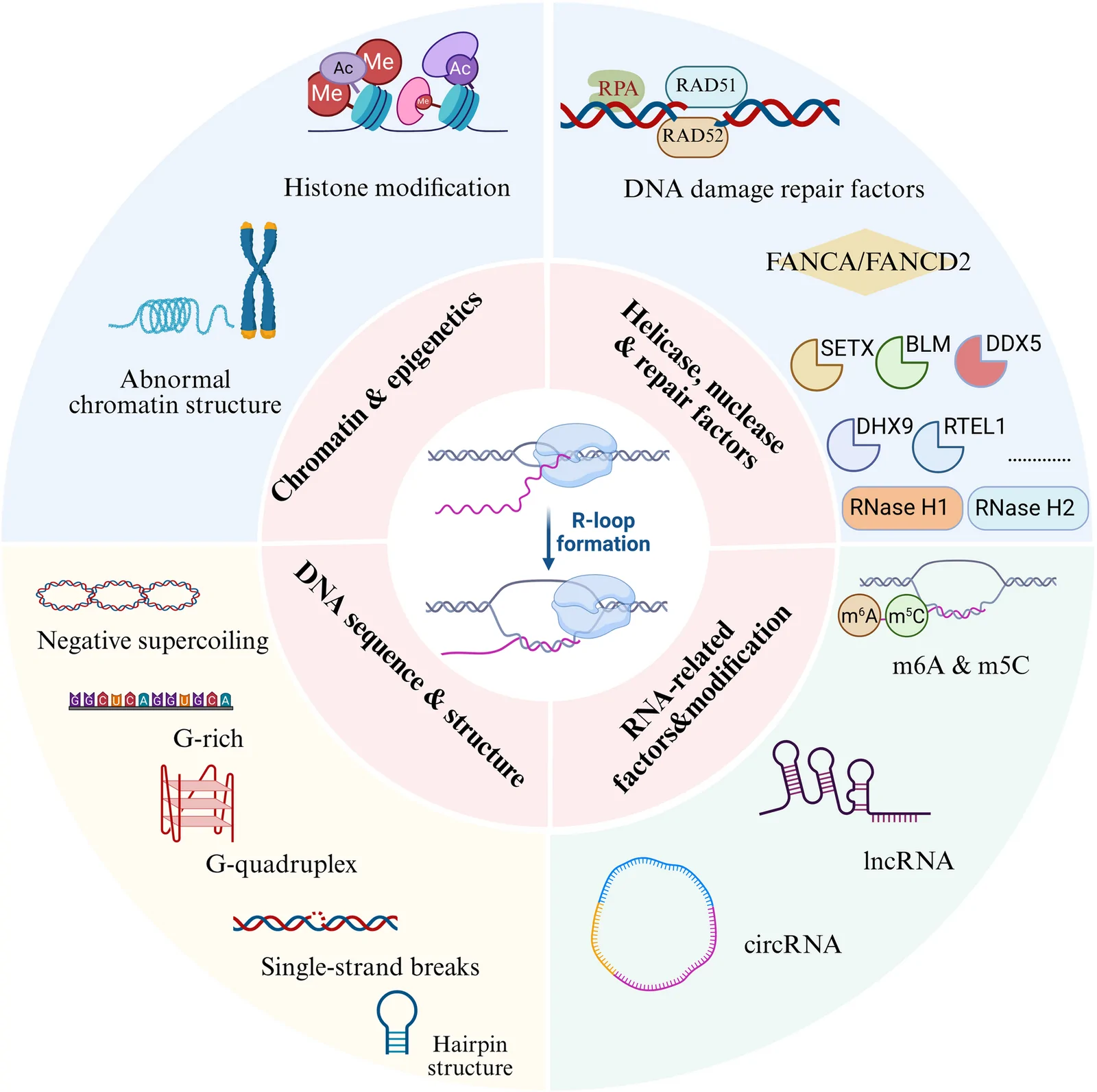
Multifactorial regulatory network governing R-loop homeostasis. Schematic depicting four interconnected regulatory axes that influence R-loop formation, stability, and resolution. The inner circle represents the R-loop itself, and the surrounding sectors illustrate the principal regulatory categories: (i) Chromatin and epigenetics, encompassing histone post-translational modifications (acetylation, methylation) and aberrant chromatin organization, both of which shape the underlying transcriptional landscape and R-loop persistence [59]; (ii) Helicases, nucleases, and repair factors, including the R-loop-resolving helicases and ribonucleases SETX, DHX9, DDX5, BLM, and RTEL1 together with RNase H1 and RNase H2, as well as DNA damage-response factors (RPA, RAD51, RAD52) and Fanconi anemia proteins (FANCA, FANCD2) that protect or process R-loop-associated lesions [90]; (iii) DNA sequence and structural features that promote or stabilize hybrid formation, including G-rich sequences, G-quadruplexes, negative supercoiling, single-strand breaks, and hairpin structures [91]; and (iv) RNA-related factors and modifications, including m6A (e.g., METTL3/YTHDC1/YTHDF2) and m5C (e.g., TRDMT1) marks on the RNA strand, and non-coding RNAs (lncRNA, circRNA) that modulate hybrid stability [92]

Despite substantial progress, several critical knowledge gaps persist. The mechanistic basis for locus specificity, which explains why some transcribed genes sustain persistent R-loops while immediately adjacent genes do not, is incompletely explained by sequence features alone. This specificity likely involves higher-order chromatin organisation, transcription factor occupancy, and local RNA processing efficiency. The quantitative contribution of the thread-back versus extended-hybrid formation mechanisms to the total R-loop burden at specific loci has not been resolved. The combinatorial logic by which multiple regulatory inputs (e.g., m6A modification, G4 stabilisation, helicase availability) are integrated to determine net R-loop stability at individual sites is largely unknown. Finally, a key unmet methodological need is the ability to detect R-loops at single-cell and single-molecule resolution in living cells without perturbation. This capability would allow the dynamic behaviour of individual R-loop events, rather than population-averaged steady states, to be studied. Addressing these gaps will require both new technologies and conceptual frameworks that integrate quantitative, predictive modeling with dynamic, single-molecule resolution (Fig. 3).

### Biological function

#### Transcriptional regulation

As a critical regulatory element in transcription, R-loops fulfill multifaceted roles in gene expression regulation. During transcription termination, the formation of R-loops assists RNA polymerase II in pausing on the template DNA strand and promotes accurate transcription termination, a function that has been validated in multiple gene transposons [93, 94]. Classic mechanistic studies have shown that R-loop formation recruits the RNA/DNA helicase SETX at the 3’ end of the gene. SETX then promotes the loading of the 5’→3’ exonuclease Xrn2, enabling it to follow and degrade the downstream transcriptional products of RNA polymerase II, thereby triggering transcriptional termination [3]. R-loops also interact functionally with G-quadruplex structures on the non-template strand. On one hand, R-loops formed during transcription facilitate G4 formation on the non-template DNA strand to enhance mRNA production; on the other hand, they can recruit the CCCTC-binding factor (CTCF), which regulates gene expression by binding to target DNA sequences [59, 95].

Furthermore, R-loops play a significant role in shaping chromatin structure. R-loops promote chromatin decompaction and transcriptional activation by excluding DNA methyltransferases and recruiting H3K4 methyltransferases, thereby protecting CpG islands from DNA methylation. During the process of immunoglobulin CSR, the formation of R-loop is a necessary step to initiate DNA double-strand breaks, thereby enabling B cells to produce different types of antibodies [4, 96]. Chun et al. found that acute global depletion of R-loops, achieved through an inducible RNase H1 expression system in mouse embryonic stem cells, led to a significant reduction in H2A.Z occupancy at active and bivalent promoters, along with increased nucleosome density [78]. This reveals a previously unrecognized role of R-loops in maintaining promoter architecture. Additionally, non-coding RNAs participate in the regulation of gene expression by modulating R-loop formation [97, 98].

#### DNA damage repair

The dual role of R-loops in DNA damage repair reflects the complexity of their biological functions [56, 99–101]. Under normal physiological conditions, R-loops can promote homologous recombination repair by serving as signaling molecules for DNA damage and acting as docking sites for repair proteins such as RPA, RAD52, and RAD51 [99, 101]. When R-loop levels fall below normal, the efficiency of both homologous recombination and non-homologous end joining repair is reduced. Upon DNA damage, R-loops can function as recruitment platforms for repair factors, guiding repair mechanisms to the damage sites [49]. Vohhodina et al. demonstrated that BRCA1 binds TERRA RNA in telomeric regions and suppresses telomeric DNA damage in an R-loop-dependent manner [102]. Additionally, m6A-methylated R-loops facilitate the recruitment of homologous recombination repair factors through recognition by YTHDC1 [79, 80].

Under pathological conditions, abnormally accumulated R-loops increase DNA strand exposure, rendering the non-transcribed strand vulnerable to damage by endogenous enzymes, leading to base alterations or strand structural abnormalities [5]. Transcription–replication conflicts (TRCs) represent a key pathological consequence of aberrant R-loop accumulation and are closely associated with genomic instability and impaired cell survival [44]. When replication and transcription occur simultaneously in the same genomic region, inappropriate regulatory mechanisms may lead to TRCs, thereby halting both processes and causing further genomic damage [90]. This phenomenon is conserved across both eukaryotic and prokaryotic cells [91]. Depending on the relative directionality of the two processes, TRCs are classified into two types: co-directional and head-on (counter-directional) collisions. In counter-directional collision, replication and transcription proceed in opposite directions, and the harmful effects are more significant. R-loops are more likely to accumulate under these conditions [43].

Brown et al. demonstrated that the RNA export and RNA degradation complexes THO and TRAMP can prevent transcription-replication conflicts, DNA breaks and CAG repetitive contractions [89]. The role of helicases in the formation and dissociation of R-loops is crucial for preventing transcription-replication conflicts [40]. The functional overlap between transcription coupled repair (TCR) and R-loop further emphasizes the close connection between these processes, with TCR components such as CSB and XPG directly involved in R-loop dissociation [73].

Furthermore, R-loop accumulation can promote replication fork stalling and transcription–replication conflicts. The Fanconi anaemia pathway constitutes a critical buffer against R-loop-dependent replication stress, as discussed in Sect.  2.2. Notably, FANCD2 interacts with RNA processing factors, including the DEAD-box helicase DDX47, to promote R-loop removal [76]. However, the precise molecular mechanism by which these FA proteins facilitate R-loop resolution remains to be determined.

#### Telomere maintenance and cellular senescence

R-loops fulfill context-dependent regulatory roles in telomere biology. Graf et al. demonstrated that TERRA-mediated R-loop formation exhibits a telomere length-dependent regulation. When telomeres shorten to a critical length, the degradation activity of nucleases such as Rat1 and RNase H2 at short telomeres is significantly reduced, leading to preferential accumulation of TERRA and its associated R-loops at short telomeres [103]. This subsequently activates the DNA damage response (DDR) and promotes the recruitment of RAD51 recombinase, initiating homology-directed repair (HDR) to delay the onset of replicative senescence [104, 105]. RAD51 and its enhancer RAD51AP1 possess non-redundant functions in TERRA R-loop formation, as depletion of either protein significantly suppresses R-loop formation at telomeres in TERRA-overexpressing cells [105, 106]. In addition, studies by In and colleagues have unveiled that TERRA R-loops can interfere with semi-conservative DNA replication, promoting telomere maintenance through break-induced replication (BIR) and PRIMPOL-dependent repair pathways [107]. These two mechanisms operate in parallel in ALT cancer cells to ensure cell survival [108].

Telomeres are highly sensitive to oxidative stress, and reactive oxygen species (ROS) can induce telomere shortening, damage, and premature cellular senescence. Nguyen et al. demonstrated that oxidative stress triggers major structural remodeling at telomeric regions, including single-strand DNA breaks, formation of internal loop DNA structures (i-loops), dissociation of the shelterin component TRF1 from telomeres, upregulation of TERRA transcription, and increased R-loop structures [109, 110]. Notably, oxidative damage-induced R-loop formation occurs not only at cis-telomeres with elevated TERRA transcription but also at trans-telomeres without increased TERRA transcription, suggesting the existence of post-transcriptional R-loop formation mechanisms. Single-molecule studies have further confirmed that TRF2 enhances the affinity of TERRA for telomeric duplex DNA and protects it from RNase H degradation, whereas TRF1 inhibits TERRA annealing and fails to provide such protection [111, 112].

In ALT cancer cells lacking telomerase, TERRA R-loops promote telomere maintenance through a unique phase separation mechanism. Xu and colleagues reported that LSD1, a histone demethylase, undergoes phase separation through RNA-binding interactions with the G-quadruplex structure of TERRA, resulting in the formation of TERRA-LSD1 condensates [113]. LSD1 localization to ALT telomeres depends on TERRA, and its function in ALT is largely independent of its demethylase activity. This mechanism provides novel insights into how the biophysical properties of histone-modifying enzyme-RNA interactions influence chromatin function. Additionally, Porro et al. subsequently demonstrated that under telomere deprotection conditions, elevated TERRA levels promote LSD1 recruitment to telomeres, where LSD1 interacts with MRE11 and enhances its nuclease activity, facilitating removal of 3′ telomeric G-overhangs [113, 114]. These findings reveal the multifaceted functions of the TERRA-LSD1 axis in ALT telomere maintenance and telomere deprotection responses.

#### Cancer development and progression

The R-loop, a three-stranded nucleic acid structure composed of an RNA-DNA hybrid and a displaced single-stranded DNA, plays a dual role in cancer development and progression [27]. Recent research indicates that dysregulation of R-loop homeostasis is a common feature in various cancers. Its aberrant accumulation can induce genomic instability, DNA damage, and transcription-replication conflicts, ultimately driving tumorigenesis [115]. In cancer-related inflammatory responses, R-loop-mediated genomic instability can activate key inflammatory signaling pathways, including the cGAS-STING axis and NF-κB signaling, thereby shaping a pro-tumorigenic inflammatory microenvironment [116, 117]. Emerging studies reveal that R-loops can also activate the type I interferon response by inducing cytoplasmic RNA-DNA hybrids, influencing the tumor immune microenvironment and therapeutic response. In hematological malignancies such as leukemia, abnormal R-loop accumulation caused by genetic mutations or environmental factors is closely associated with genomic instability, serving as a significant driver of disease pathogenesis [118]. R-loops at different genomic loci exhibit functional heterogeneity: telomeric R-loops participate in the ALT mechanism in ALT-positive cancer cells; R-loops in the nucleolar rDNA region can trigger nucleolar stress to induce tumor cell death, with nucleolar R-loop levels serving as a potential biomarker for stratifying ribosome-dependent cancers and demonstrating prognostic value in colorectal cancer [119]; R-loops at centromeric and other non-canonical sites affect chromosomal stability through distinct mechanisms [120].

R-loop scoring models show great potential for prognostic assessment and treatment response prediction across multiple cancers. In lung adenocarcinoma, scoring systems based on R-loop-associated genes effectively predict overall patient survival, immune features, and drug sensitivity, with key genes like EIF3B, ALDOA, and IGF2BP1 validated as potential biomarkers [121]. Single-cell RNA sequencing studies reveal that low R-loop scores correlate with activated glycolysis, epithelial-mesenchymal transition, immune evasion, and therapy resistance, whereas high R-loop scores are associated with advanced stages, immunosuppressive microenvironments, and poor prognosis. In hepatocellular carcinoma, R-loop scoring models can effectively assess the tumor immune microenvironment, lipid metabolism reprogramming, and prognosis [122]. CLTC is identified as a key R-loop regulatory gene, and targeting CLTC can inhibit R-loop-mediated lipid metabolic reprogramming, suppressing tumor proliferation and metastasis. In osteosarcoma, a machine learning-based R-loop Gene Prognostic Scoring Model (RGPSM) can predict patient prognosis, Huvos grading, and tumor microenvironment characteristics, with PSIP1 identified as a key R-loop-related gene promoting osteosarcoma progression [123]. Multiple studies have confirmed the prognostic value of R-loop scores across 33 cancer types, and their ability to predict patient responses to targeted therapy, chemotherapy, and immunotherapy in 32 independent cohorts.

Regarding therapeutic strategies, approaches targeting R-loops include: (i) Promoting R-loop accumulation to induce cancer cell death, such as inducing R-loops in the nucleolar rDNA region to trigger nucleolar stress. (ii) Inhibiting R-loop resolution to exacerbate genomic instability, particularly in tumors with homologous recombination deficiency [124]. (iii) Combining with m⁶A modification regulation. METTL3 inhibitors (e.g., STM3006, STC-15) can affect R-loop homeostasis, triggering double-stranded RNA formation, activating intracellular interferon responses, and enhancing anti-tumor immunity, some of which have entered clinical trials [125, 126]. However, employing R-loops as biomarkers still faces numerous challenges, including the need to develop more specific and effective targeted inhibitors; establishing standardized R-loop detection techniques (e.g., DRIP-seq, liquid biopsy biosensors) [127, 128]; and identifying cancer subtypes suitable for specific therapies.

#### Neurodegenerative diseases

R-loop dysregulation is closely associated with the pathogenesis of multiple neurodegenerative diseases, including Amyotrophic Lateral Sclerosis (ALS), Spinal Muscular Atrophy (SMA), ataxias, Huntington’s disease, and Short Tandem Repeat (STR) expansion disorders such as Friedreich’s ataxia [129, 130]. High transcriptional activity in neurons increases R-loop levels, and failure to resolve them promptly leads to accumulated DNA damage and neurodegeneration. In SMA, mutations in the SMN1 gene lead to reduced SMN protein levels, causing downregulation of ZPR1 and SETX and decreased DNA-PKcs activity, ultimately resulting in abnormal R-loop accumulation, increased DNA double-strand breaks, and neuronal death [130, 131]. Interestingly, different diseases exhibit opposing alterations in R-loop metabolism: SMA shows high-level R-loop accumulation, whereas ALS4 (caused by SETX gene mutations) demonstrates reduced R-loop levels, suggesting that SETX mutations lead to a gain-of-function alteration. In ALS10, TDP-43 depletion or mutation leads to increased R-loops and DNA damage accumulation, associated with defects in DNA-PKcs-mediated non-homologous end joining (NHEJ) repair [132]. In STR expansion disorders, R-loop formation increases with repeat sequence length and promotes repeat instability, driving disease progression and intergenerational anticipation. SETX, an ATP-dependent RNA/DNA helicase, plays a central role in R-loop resolution, and its dysfunction underlies the pathogenesis of Ataxia with Oculomotor Apraxia type 2 (AOA2) and ALS4.

### Detection methods

Accurate detection of R-loops is crucial for understanding their biological functions and disease relevance. The mainstream methods primarily include immunoprecipitation sequencing techniques based on the S9.6 antibody (e.g., DRIP-seq and its variants), though the S9.6 antibody has limitations due to cross-reactivity with double-stranded RNA [63, 133, 134]. To overcome this, methods utilizing the hybrid binding domain (HBD) of RNase H1 (e.g., R-ChIP and MapR) offer improved specificity [81, 135–137]. A key methodological advance came in 2025 with the development of RIAN-seq, an antibody-free technique that achieves base-pair resolution by selectively digesting non-hybrid nucleic acids, demonstrating vastly superior sensitivity and resolution compared to previous methods [138]. Additionally, live-cell imaging technologies (e.g., the RHINO sensor) and single-molecule analyses (e.g., DNA combing coupled with immunofluorescence) enable in situ visualization of R-loop dynamics [44, 139, 140]. Complementary approaches such as chemical labeling methods (e.g., spKAS-seq) and deep learning-based prediction tools further enrich the methodological toolkit [141, 142]. The integration of these diverse technologies continues to refine our mechanistic understanding of R-loop formation and its physiological and pathological functions.

## D-loop

### Basic structure

D-loops (Displacement loops) are unique three-stranded nucleic acid structures characterized by the separation of two complementary strands within a double-stranded DNA molecule over a defined region, wherein a third DNA strand invades and pairs with one of the separated strands through Watson-Crick base pairing, thereby displacing the other strand to form a single-stranded loop structure [143]. Morphologically, D-loops resemble the letter “D,” with the displaced single-stranded DNA forming a protruding loop while the invading third strand forms a heteroduplex DNA (hDNA) region with the template strand. Based on biological function and genomic location, D-loops can be classified into three major types: (i) mitochondrial D-loops [143]; (ii) homologous recombination D-loops [144]; and (iii) D-loops within telomeric T-loops [24, 145].

#### Mitochondrial D-loops

Mitochondrial D-loops reside within the major non-coding region (NCR) of the mitochondrial genome, representing a relatively stable three-stranded DNA structure [146, 147]. The human mitochondrial DNA is a circular double-stranded molecule of approximately 16.5 kb, with the NCR spanning roughly 1.1 kb. Within this region, a short DNA strand of approximately 650 nucleotides (termed 7 S DNA) hybridizes with the parental light strand (L-strand), simultaneously displacing the parental heavy strand (H-strand) to form the classical three-stranded D-loop structure [148, 149].

**Fig. 4:**
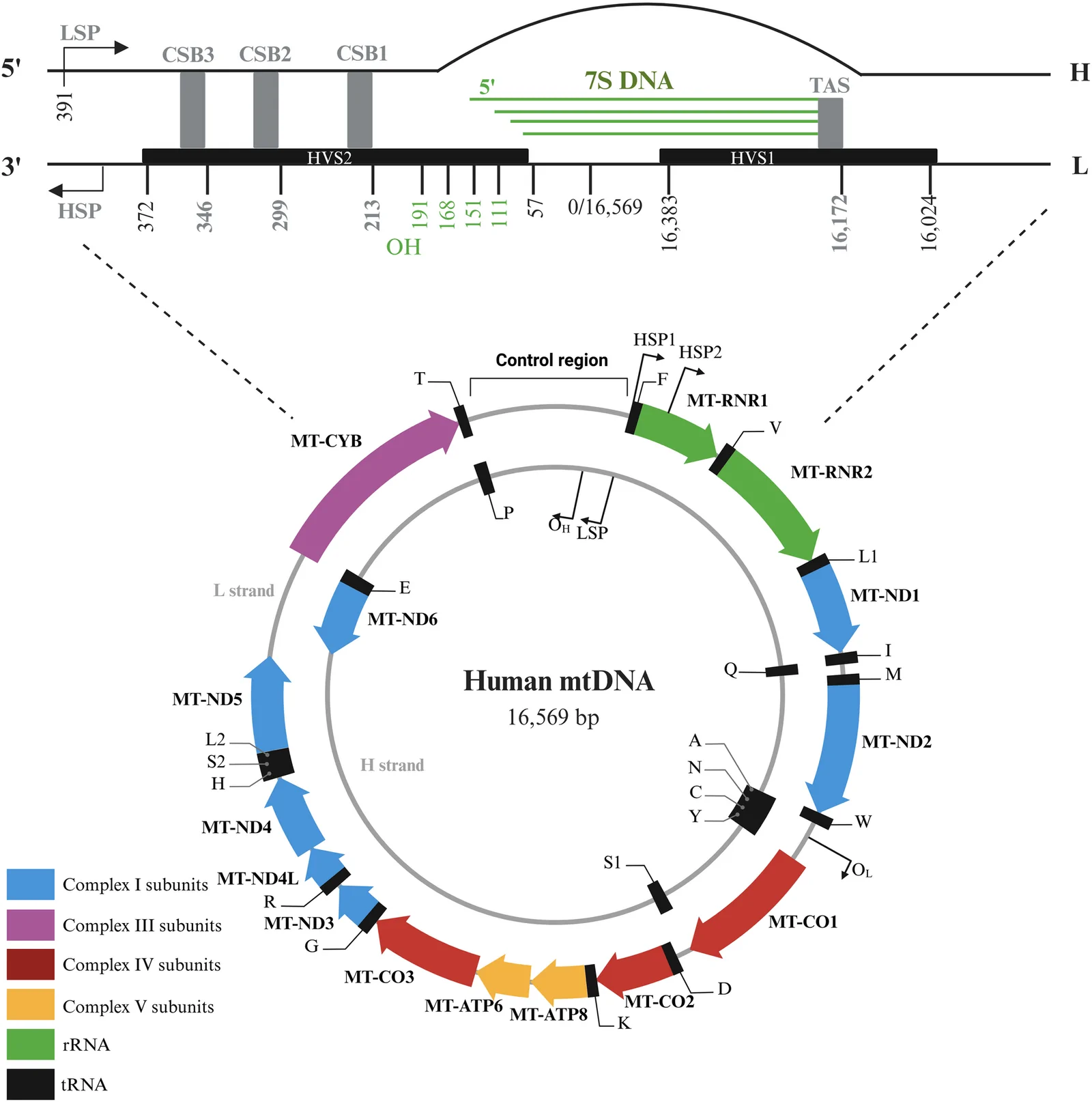
Structural organization of the human mitochondrial genome and its D-loop region. (Top) Schematic of the major non-coding region (NCR) and D-loop of human mtDNA [143]. The 5’ ends of 7 S DNA are shown in green, together with the heavy-strand origin of replication (OH), the heavy- and light-strand promoters (HSP, LSP), the conserved sequence blocks CSB1–CSB3, the termination-associated sequence (TAS), and the hypervariable segments HVS1 and HVS2. The displaced strand of the D-loop is depicted as the arc above the parental duplex. (Bottom) Circular map of the 16,569 bp human mtDNA [153]. Genes encoding subunits of respiratory complex I (MT-ND1–6, MT-ND4L; blue), complex III (MT-CYB; purple), complex IV (MT-CO1–3; red), and complex V (MT-ATP6/8; orange) are indicated, along with the two mitochondrial rRNAs (MT-RNR1, MT-RNR2; green) and 22 tRNAs (black, single-letter amino acid code) distributed along the heavy (H) and light (L) strands [154]. The non-coding control region harbors OH, the light-strand origin of replication (OL), HSP1/HSP2, and LSP [155]

The mitochondrial D-loop region harbors critical regulatory elements, including the heavy-strand origin of replication (OH), the heavy-strand promoter (HSP), and the light-strand promoter (LSP), thereby playing a central role in regulating mitochondrial DNA replication and transcription [150, 151]. The mitochondrial D-loop region is the most hypervariable locus in the mitochondrial genome, with a mutation rate approximately 5–10 times higher than other mitochondrial coding regions [152] (Fig. 4).

#### Homologous recombination D-loops

**Fig. 5:**
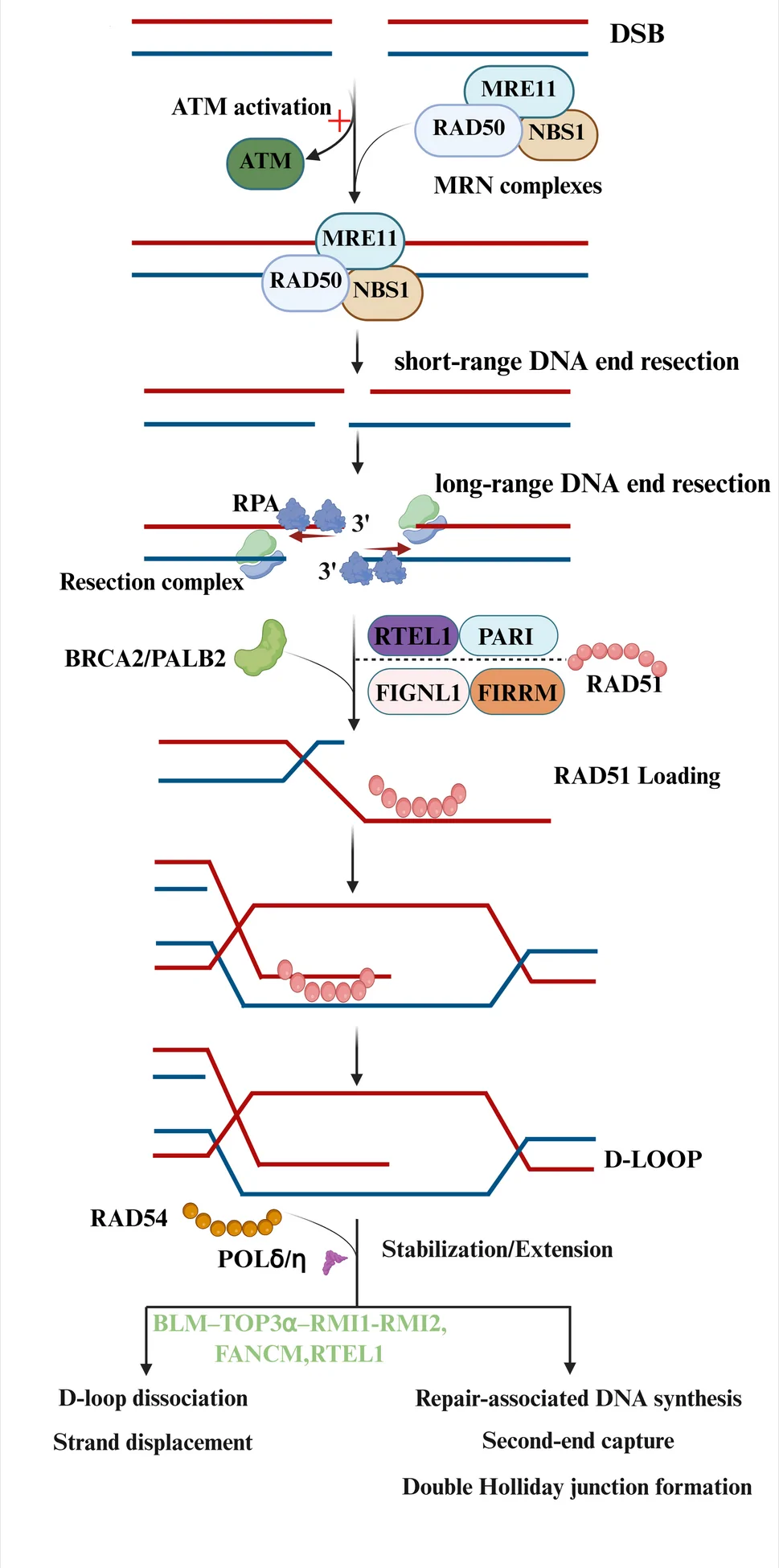
Molecular pathway of D-loop formation during homologous recombination-mediated DSB repair. Following a DSB, the MRN complex (MRE11–RAD50–NBS1) recognizes and tethers the broken DNA ends, activating the ATM kinase to initiate the DNA damage response [160]. MRE11 endonuclease activity, stimulated by CtIP, introduces a nick near the DSB, followed by short-range 3′→5′ exonucleolytic processing by MRE11/CtIP to generate an initial ssDNA substrate [161]. This is a prerequisite for the subsequent long-range 5′→3′ resection carried out by EXO1 or the BLM–DNA2 axis, both acting as part of the resection complex (EXO1- or DNA2-containing), to produce extensive 3′ ssDNA overhangs. The resulting ssDNA is rapidly coated by RPA to form a nucleoprotein filament. The mediator complex BRCA2/PALB2 facilitates displacement of RPA and loads RAD51 onto ssDNA, assembling the presynaptic RAD51 filament. RAD51 filament dynamics are tightly regulated: anti-recombinases including RTEL1, PARI, and the FIGNL1–FIRRM (FLIP) complex actively dismantle RAD51 filaments to maintain HR fidelity and prevent aberrant recombination [162]. The mature presynaptic filament drives homology search and strand invasion into the intact homologous duplex, generating the D-loop intermediate. RAD54 stabilizes the D-loop and remodels chromatin, while DNA polymerases δ and η (POLδ/η) extend the invading strand to initiate repair-associated DNA synthesis. The D-loop intermediate can be further processed via second-end capture and double Holliday junction (dHJ) formation to complete conservative repair. Alternatively, the D-loop undergoes dissociation and strand displacement mediated by the BLM–TOP3α–RMI1–RMI2 dissolvasome and FANCM, suppressing aberrant joint molecules and limiting crossover formation

Homologous recombination D-loops represent critical intermediate structures during the repair of DNA double-strand breaks via HR [156]. Following DSB formation, the broken ends undergo 5’ to 3’ exonucleolytic resection mediated by the MRE11-RAD50-NBS1 (MRN) complex and additional nucleases such as EXO1 and DNA2, generating extensive 3’ single-stranded DNA (ssDNA) overhangs [157]. These ssDNA overhangs are subsequently coated by the recombinase RAD51 (in eukaryotes) or RecA (in prokaryotes) to form helical nucleoprotein filaments, which then catalyze homology search and strand invasion into homologous double-stranded DNA templates, establishing D-loop intermediates [158]. Structural features of homologous recombination D-loops include the heteroduplex DNA region formed between the invading strand and the complementary template strand, the displaced single strand forming the characteristic loop, and the strand exchange junctions at both termini [159] (Fig. 5).

#### Telomeric D-loops

Telomeric D-loops form when the 3’ G-rich single-stranded overhang (typically 50–400 nucleotides in mammals) at chromosome termini invades upstream double-stranded telomeric repeat sequences (TTAGGG in vertebrates), creating a lariat-like higher-order structure termed the T-loop (Telomere loop) [163]. At the base of the T-loop, where the 3’ overhang hybridizes with the double-stranded DNA through strand invasion, a canonical D-loop structure is established. This higher-order architecture is mediated primarily by the telomere-binding protein TRF2 (Telomeric Repeat-binding Factor 2) and effectively sequesters chromosome ends, preventing their inappropriate recognition as DNA breaks by the DDR pathway [164, 165].

**Fig. 6:**
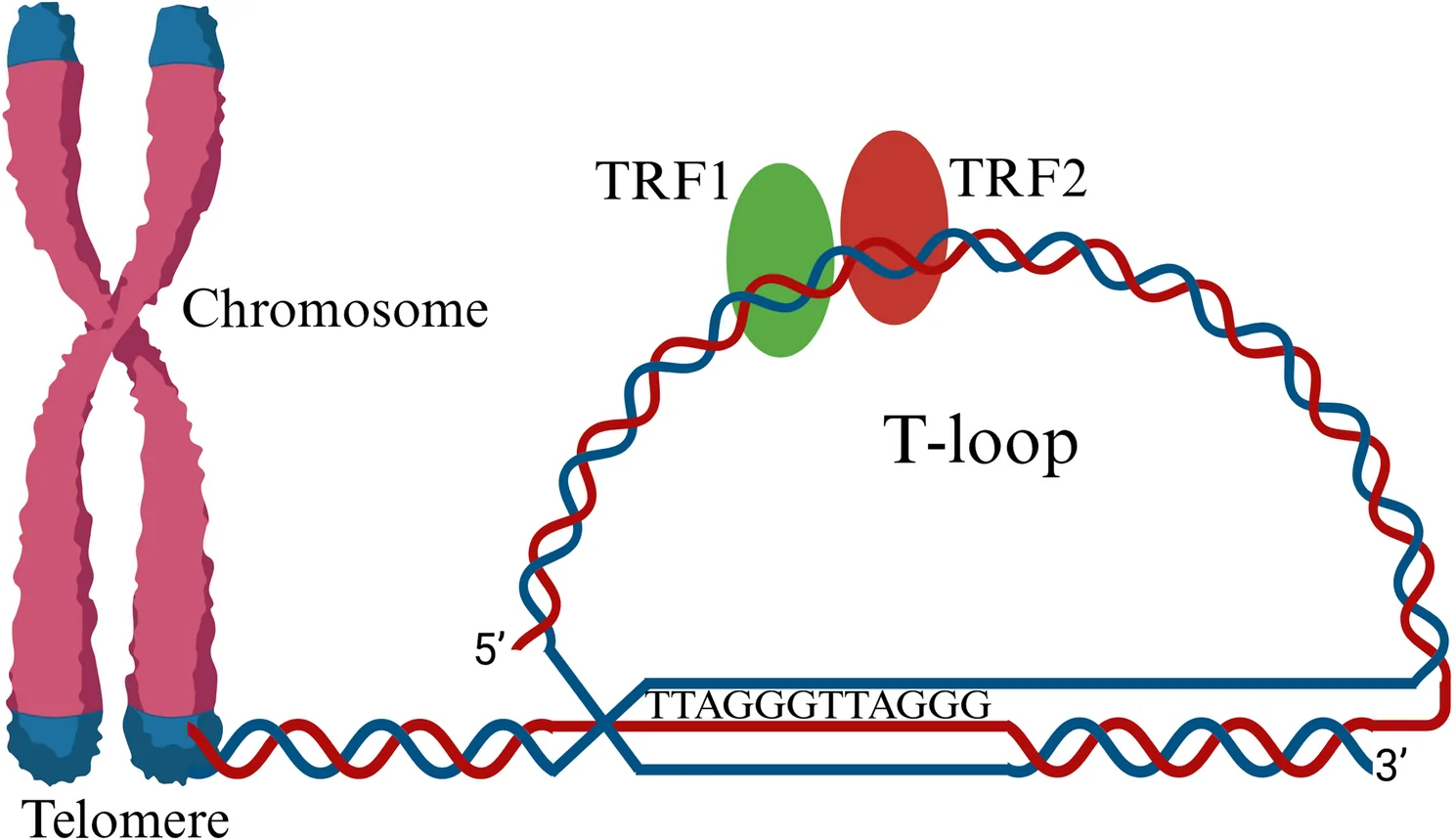
Architecture of the telomeric D-loop (T-loop) and shelterin-mediated stabilization [169]. Schematic of telomere end protection at a single chromosome terminus. The duplex telomeric DNA (TTAGGG repeats; the two complementary strands shown in red and blue) folds back on itself so that the 3′ G-rich single-stranded overhang invades the proximal double-stranded telomeric repeats of the same chromosome end, base-pairing with the C-rich strand and displacing the G-rich strand. This intramolecular strand invasion generates a large lariat-shaped T-loop together with a displacement loop (D-loop) at the invasion junction [170]. The double-stranded telomeric repeats are bound by the shelterin proteins TRF1 (green) and TRF2 (red); TRF2 in particular promotes T-loop formation and protects the D-loop junction, providing the architectural scaffold required for T-loop maintenance. By sequestering the linear chromosome end within this higher-order intramolecular structure, the T-loop prevents its inappropriate recognition as a double-strand break, thereby suppressing activation of the ATM/ATR-dependent DNA damage response and end-to-end chromosomal fusions. The 5′ and 3′ ends of the telomeric strands are indicated

The T-loop structure represents an effective molecular solution to the “end-protection problem,” as it sequesters the chromosome terminus from upstream DNA damage sensors (e.g., the MRN complex and RPA‑ATRIP) that recruit the downstream signaling kinases ATM and ATR, respectively, while maintaining telomere stability and preventing end-to-end chromosome fusions [166]. Recent high-resolution structural studies combining cryo-electron microscopy and atomic force microscopy have demonstrated that tetrameric TRF2, in conjunction with its binding partner RAP1, is essential for T-loop formation and stabilization, with TRF2’s architectural properties and DNA-bending capacity directly facilitating the DNA strand invasion required for D-loop establishment [167]. Furthermore, the shelterin complex, comprising TRF1, TRF2, POT1, TIN2, TPP1, and RAP1, collaboratively regulates T-loop dynamics and telomere protection [168] (Fig. 6).

### Regulation of formation and stability

D-loop structures, despite sharing a common three-stranded topology, arise through fundamentally distinct molecular mechanisms depending on their biological context: HR-mediated D-loops, telomeric D-loops embedded within T-loops, and mitochondrial D-loops coupled to mtDNA replication. Understanding the formation and stability of each class requires not only a mechanistic inventory of the proteins involved, but also a critical appreciation of how competing regulatory forces are balanced in space and time. While substantial progress has been made in delineating individual regulatory factors, the field faces two persistent conceptual tensions: (i) how the same core recombination machinery can generate both genome-stabilizing and genome-destabilizing outcomes; and (ii) how physiological and aberrant D-loops are differentially recognized at different genomic loci. These unresolved questions underscore that our current models remain incomplete.

#### Sources of D-loop formation

##### Mitochondrial D-loops: replication-coupled origin

Mitochondrial D-loops occupy a unique niche as stable, replication-associated structures rather than transient repair intermediates. According to the strand-displacement replication model, which is the most widely accepted but still debated framework, mitochondrial DNA replication initiates within the non-coding control region (NCR). In this model, transcription from the light-strand promoter (LSP) produces an approximately 200-nucleotide RNA (7 S RNA) that remains stably base-paired with the template L-strand [151, 171]. This RNA–DNA hybrid, stabilized by G-quadruplex structures near Conserved Sequence Block 1 (CSB1), serves as a primer for DNA synthesis by the mitochondrial DNA polymerase γ (POLγ) [172].

POLγ, a heterotrimeric complex of one catalytic POLγA subunit and two accessory POLγB subunits, synthesizes a nascent H-strand using 7 S RNA as a primer. The TWINKLE helicase facilitates strand unwinding ahead of the polymerase. Synthesis proceeds until the Termination-Associated Sequence (TAS), producing an approximately 650-nucleotide 7 S DNA fragment [173, 174]. This fragment remains base-paired with the parental L-strand while displacing the H-strand to form the three-stranded D-loop. With a half-life of approximately one hour, 7 S DNA undergoes continuous turnover. This establishes the mitochondrial D-loop as a dynamically maintained structure in thermodynamic equilibrium, a property that distinguishes it sharply from the stable telomeric D-loop and the transient HR D-loop.

It is worth noting that the strand-displacement model has been challenged by the RITOLS (Ribonucleotide Incorporation ThroughOut the Lagging Strand) model and the strand-coupled replication model, which propose distinct initiation mechanisms and origins [175]. The relative contributions of these models to steady-state mtDNA maintenance versus stress-induced replication remain an active area of debate. Furthermore, a major replication origin at position 57 within the D-loop of human cells has recently been identified. Nascent chains from this origin proceed beyond the classical 3′ end of the D-loop, suggesting the existence of at least two mechanistically distinct modes of mtDNA synthesis [176]. These observations challenge the traditional view of TAS as an absolute replication termination checkpoint and call for more nuanced models of mitochondrial D-loop origin.

##### Homologous recombination–associated D-loops

The canonical source of HR D-loops is the repair of DNA double-strand breaks [177, 178]. Following DSB induction, the broken ends undergo 5′-to-3′ resection mediated by the MRN complex, CtIP, and long-range nucleases (EXO1 or DNA2–BLM/WRN), generating 3′ single-stranded DNA (ssDNA) overhangs rapidly coated by RPA [179–181]. RPA protects ssDNA from nucleolytic attack and secondary structure formation but must be displaced before the critical recombinase loading step. Recombination mediators, principally BRCA2 in mammals and RAD52 in yeast, catalyze RPA eviction and nucleate RAD51 onto ssDNA, forming the right-handed helical presynaptic filament [182, 183]. This filament constitutes the active effector that searches the genome for homologous sequences and catalyzes strand invasion into the donor duplex, physically displacing one strand to create the displacement loop [184].

Importantly, D-loops do not arise exclusively from DSBs. Single-ended DSBs at collapsed replication forks, inter-strand crosslinks processed into one-ended breaks, and programmed DSBs during meiosis (catalyzed by SPO11) represent alternative and physiologically critical sources of HR D-loops [185, 186]. The mechanistic differences between these initiation events, particularly in terms of resection length, recombinase identity (RAD51 vs. DMC1 in meiosis), and template availability, likely produce D-loops with distinct structural and kinetic properties. However, direct comparative studies remain sparse. This represents an underappreciated source of variation in D-loop behavior across cell types and cell cycle stages.

Recent cryo-electron microscopy studies have transformed our mechanistic understanding of D-loop formation. Structural analyses captured five distinct intermediates during RAD51-mediated strand exchange, revealing that the N-terminal domain (NTD) recruits and bends donor dsDNA while the L2 loop promotes strand separation and stabilizes the nascent heteroduplex [14]. Complementary structural work by Luo et al. unveiled a RAD51–ADP double filament that undergoes a synergistic mechanical collapse, linking nucleotide state to filament integrity and providing a structural basis for filament disassembly after D-loop formation [14]. Collectively, these findings establish D-loop formation as a highly coordinated, stepwise process rather than a single concerted reaction. This conceptual advance has important implications for understanding why some D-loops are efficiently extended while others are rapidly reversed.

##### Telomeric D-loops within T-loops

At chromosome termini, the 3′ G-rich single-stranded overhang (50–400 nucleotides in mammals) invades upstream double-stranded telomeric repeats (TTAGGG), generating a lariat-like architecture termed the T-loop, within which a canonical D-loop is embedded [187]. This structure was initially inferred from electron microscopy images and has since been directly visualized by atomic force microscopy and cryo-electron tomography. The telomeric D-loop differs fundamentally from its HR counterpart in that it is designed for structural persistence rather than transient repair—it must be maintained throughout interphase to mask chromosome ends from the DDR, yet must be resolved during S phase to allow telomere replication [145, 163].

A mechanistic distinction that is often underappreciated is that telomeric D-loop formation does not require a classical recombinase such as RAD51; instead, it is driven primarily by the architectural properties of TRF2, which promotes DNA bending, strand invasion, and loop stabilization [188].

#### Factors regulating D-loop formation and stability

D-loop regulation operates through multiple, partially overlapping layers that together determine whether a D-loop is formed, extended, maintained, or dissolved. It is analytically useful to organize these factors by their primary mode of action, but it must be emphasized that these layers are highly interdependent: changes in DNA topology alter recombinase filament dynamics; cell cycle position gates helicase activities; post-translational modifications remodel protein–protein interactions across all layers simultaneously.

##### DNA topology and supercoiling

Negative supercoiling, which results from underwound duplex tension, represents the most fundamental biophysical prerequisite for efficient D-loop formation. By facilitating local strand separation, negative supercoiling directly reduces the energetic cost of third-strand pairing. RecA-catalyzed D-loop formation on negatively supercoiled substrates is substantially more efficient than on relaxed DNA in vitro [189, 190]. In eukaryotes, RAD54 generates unconstrained negative supercoils behind its translocase path through ATP hydrolysis-driven DNA translocation. These topological perturbations are specifically exploited by RAD51 presynaptic filaments to promote synapsis and heteroduplex extension [191].

Global superhelical density is modulated by topoisomerases. In humans, this enzyme family comprises six members that act on a broad range of DNA and RNA substrates in both nuclear and mitochondrial compartments: the type IB enzymes TOP1 and the dedicated mitochondrial isoform TOP1MT (also referred to as mtTOP1), the type IIA enzymes TOP2A and TOP2B, and the type IA enzymes TOP3A and TOP3B [192]. By relaxing transcription-induced supercoiling, these enzymes indirectly influence D-loop propensity at specific genomic loci, particularly in transcriptionally active regions where torsional stress is most pronounced. Beyond this global regulatory role, TOP3A and TOP3B exert direct, substrate-specific effects on recombination and transcription-associated nucleic acid structures. As the catalytic component of the BTRR (BLM-TOP3A-RMI1-RMI2) dissolvasome, TOP3A directly disrupts RAD51-mediated D-loops and dissolves double Holliday junctions through its type IA strand-passage activity, thereby acting as a key determinant of D-loop turnover and homologous recombination outcomes [193, 194]. In contrast, TOP3B, the only RNA topoisomerase in metazoans, resolves R-loops in coordination with the DEAD-box helicase DDX5 by cleaving the displaced single-stranded DNA of the R-loop, thereby suppressing R-loop-associated replication stress and genome instability [195]. In the mitochondrial genome, which is a topologically constrained circular DNA, supercoiling status is dynamically coupled to LSP transcription initiation and TAS-mediated replication termination. TOP1MT and the mitochondrially imported isoform of TOP3A together maintain mtDNA topological homeostasis during replication and transcription [196], creating a local topological microenvironment that is integral to D-loop maintenance [151]. Despite its fundamental importance, the quantitative relationship between supercoiling state and D-loop frequency at specific genomic loci in living cells remains technically inaccessible with current methods [197]. This represents an important gap, given that supercoil-mediated regulation may explain the locus-selectivity of HR [198, 199].

##### RAD51/RecA nucleoprotein filament dynamics

The intrinsic properties of the RAD51–ssDNA nucleoprotein filament are central determinants of D-loop formation efficiency and outcome. ATP-bound RAD51 assembles into a stiff, right-handed helical filament with three nucleotides per monomer, stretching ssDNA to ~ 150% of its B-form length and creating a conformation permissive for dsDNA insertion and homology interrogation [184]. Homology recognition occurs through direct physical contact between dsDNA and the presynaptic filament without requiring complete duplex separation. The double helix unwinds only after homologous sequences are identified [200, 201]. This paradigm shift, supported by biochemical, NMR, and single-molecule studies, has significant implications. It suggests that the initial synaptic complex is kinetically reversible and that D-loop stability is not automatically guaranteed once the filament contacts duplex DNA.

ATP hydrolysis by RAD51 drives filament disassembly and is tightly regulated to prevent persistent, ectopic filaments. The RAD51 ADP-bound double filament, resolved by cryo-EM, represents a collapsed, inactive conformation that may serve as a structural intermediate during filament turnover. RAD51 paralogs (RAD51B, RAD51C, RAD51D, XRCC2, and XRCC3) assemble into two subcomplexes, BCDX2 and CX3. These complexes facilitate RAD51 recruitment and stabilization at different stages of D-loop formation [202]. Two recent cryo-EM studies have substantially refined this picture: the paralogs in fact form two heterotetrameric assemblies — the RAD51B complex (RAD51B–RAD51C–RAD51D–XRCC2) that drives ATP-hydrolysis-dependent nucleation of RAD51 filaments, and the XRCC3 complex (XRCC3–RAD51C–RAD51D–XRCC2) that stably caps the 5′ termini of RAD51 filaments to promote homologous pairing [203]; furthermore, all five paralogs can co-assemble into higher-order BCDX2–CX3 and DX2–CX3 supercomplexes that act, respectively, as a dynamic “loader” templating RAD51 filament nucleation and growth, and as a stable “anchor” that secures the assembled filament on ssDNA [204]. The meiosis-specific recombinase DMC1 employs analogous but architecturally distinct filaments. These preferentially promote interhomolog strand invasion, whereas RAD51 mediates intersister repair. This distinction has direct implications for crossover versus non-crossover outcome determination [205]. A persistent challenge in the field is that filament dynamics measured in vitro may not accurately reflect behavior in the chromatin context. In vivo, nucleosome positioning, histone modifications, and chromatin-remodeling complexes impose additional constraints on filament assembly and disassembly.

##### Pro-recombination factors promoting D-loop formation and extension

BRCA2 is the paramount positive regulator of D-loop formation in mammals. Its mediator activity is distributed across structurally and functionally distinct modules. The eight BRC repeats serve as the principal RAD51-binding platform: they engage RAD51 monomers, modulate the nucleotide-state and DNA-binding selectivity of RAD51, suppress unproductive RAD51 association with dsDNA, and channel RAD51 onto resected ssDNA. The co-crystal structure of the BRC4 motif bound to a RAD51 fragment provided the molecular basis for this interaction [206, 207]. Displacement of RPA from ssDNA is driven not by the BRC repeats themselves, but chiefly by the BRCA2 DNA-binding domain (DBD) acting in cooperation with its tightly associated partner DSS1. DSS1 structurally and electrostatically mimics ssDNA, remodels the RPA-ssDNA interface, and thereby licenses RAD51 nucleation onto resected ends [208]. A topologically distinct C-terminal RAD51-binding region, the TR2 (CTRD) domain, then stabilizes the assembled presynaptic filament against disassembly by competing BRC interactions, an activity that is negatively regulated by CDK-dependent phosphorylation of Ser3291 as cells enter mitosis [209, 210]. Structural analysis revealed that the BRCA2 C-terminus induces a unique ATP–RAD51 dimer conformation that accommodates nucleation directly onto double-stranded B-DNA, providing mechanistic insight into BRCA2’s role in replication fork protection independent of canonical HR [211]. PALB2 bridges BRCA1 and BRCA2, enabling damage-site recruitment of the holocomplex, and BRCA1 additionally counteracts 53BP1-mediated end protection to license resection and RAD51 loading in S/G2 phase [212, 213].

RAD54 (Rad54 in *Saccharomyces cerevisiae*; RAD54L in mammals), a Snf2-family DNA translocase, functions as a multifaceted co-factor at multiple stages of D-loop formation. It generates negative supercoils that stimulate presynaptic filament activity, promotes heteroduplex extension by pumping newly synthesized DNA into the D-loop, and removes RAD51 from post-synaptic heteroduplex DNA. This latter activity is essential for primer–template formation and subsequent DNA synthesis [214]. Rad54 exists as both monomers and higher-order oligomers (hexamers or dodecamers) on DNA, and its phosphorylation status critically determines the balance between strong DNA binding and dynamic translocation [215]. A conserved latch domain within Rad54 stabilizes early strand invasion intermediates; mutations in this domain reduce translocation velocity and paradoxically increase crossover frequencies, demonstrating that the kinetic properties of Rad54 are as important as its catalytic activity for pathway outcome [216].

RAD52 plays a partially redundant role with BRCA2 in RAD51 loading, and is particularly important for single-strand annealing and second-end capture at D-loop intermediates. FIGNL1 and its binding partner FIRRM restrict inappropriate RAD51 loading, particularly during meiosis, by promoting RAD51 disassembly from chromatin outside of defined repair windows [217]. The HROB (MCM8IP) protein and its associated helicase MCM8–MCM9 promote RAD51 filament extension and D-loop formation in response to inter-strand crosslinks and replication fork stalling [218]. Collectively, these pro-recombination factors form a layered assembly line for D-loop construction. However, their precise ordering, redundancy, and context-dependence remain incompletely defined. This is particularly true in non-dividing cells and during the DNA damage response outside canonical S/G2 phase.

##### Anti-recombination factors and D-loop disassembly

The homeostasis of D-loops is critically maintained by a network of helicases and anti-recombination factors that actively dismantle these structures, preventing their conversion into deleterious recombination intermediates. Srs2 in yeast functions as the primary anti-recombinase: its interaction with PCNA at replication forks enables it to selectively dismantle RAD51 filaments on duplex DNA, channeling repair toward PCNA-dependent synthesis-dependent strand annealing rather than crossover-producing HR [219]. The Sgs1–Top3–Rmi1 complex, orthologous to BLM–TOP3α–RMI1 in mammals, resolves D-loops and Holliday junction intermediates through a dissolution mechanism that strictly avoids crossover formation. This is a key distinction from structure-specific nucleases that cleave HJs [220].

RTEL1 (Regulator of Telomere Elongation Helicase 1) occupies a particularly important position as it antagonizes D-loops in two distinct contexts: it unwinds HR D-loops genome-wide to enforce SDSA (Synthesis-Dependent Strand Annealing) over crossover pathways, and it is specifically recruited by TRF2 to T-loops during S phase to permit telomeric replication [169, 221]. Mph1 (FANCM in mammals) disrupts D-loop intermediates and thereby prevents multi-invasion (MI) events, in which a single Rad51-coated ssDNA filament simultaneously invades two distinct donor duplexes. As first defined and mechanistically characterized in budding yeast, MI joint molecules are byproducts of the homology-search reaction that, when processed by structure-selective endonucleases, drive multi-invasion-induced rearrangements (MIR), a recombination-based mutagenic pathway that translocates otherwise intact donor loci and amplifies the original DNA damage [17, 222]. Critically, a recent study demonstrated that transcriptional activity at the donor locus can suppress D-loop formation in cis through a mechanism independent of RNA transcripts, RNA: DNA hybrids, or canonical transcription factors. This suppression is potentially more potent than that mediated by the yeast Sgs1–Top3–Rmi1 complex (BLM–TOP3α–RMI1/RMI2 in mammals), Srs2 (functionally analogous to mammalian PARI/RTEL1), or Mph1 (FANCM in mammals) individually [223]. This finding reveals an unexpected regulatory circuit connecting transcription elongation to recombination suppression, and raises important questions about how actively transcribed genes are protected from ectopic recombination at their own sequences.

RADX, a human-specific ssDNA-binding protein, counterbalances BRCA2 at stalled replication forks by stimulating RAD51 ATPase activity and destabilizing presynaptic filaments [224]. ZGRF1 promotes RAD51 removal following D-loop formation to facilitate DNA synthesis by the extension polymerase. Checkpoint kinase RAD53 (CHK2 in mammals) phosphorylates recombination factors at D-loop intermediates, imposing temporal control over recombination progression [225]. An important but unresolved question concerns the coordination among these disassembly factors: current evidence suggests functional redundancy, but whether distinct factors are preferentially recruited under different genotoxic conditions, cell cycle phases, or chromatin environments remains to be established. This distinction matters clinically, as perturbations of specific disassembly pathways have different consequences for HR fidelity and genome stability.

##### Shelterin-mediated regulation of telomeric D-loops

The telomeric D-loop, embedded within the T-loop architecture, is subject to a highly specialized regulatory system centered on the shelterin complex (TRF1, TRF2, POT1, TIN2, TPP1, RAP1). TRF2 is the principal architect of T-loop formation: its tetrameric organization imposes DNA bending, facilitating strand invasion by the 3′ overhang, while its N-terminal basic domain directly stabilizes the D-loop and protects it from BLM-mediated unwinding and RPA displacement [226]. The TRF2–RAP1 complex paradoxically recruits BLM helicase via the TRF2 TRFH domain, channeling BLM activity specifically toward unwinding D-loops within T-loops to limit RAD51-dependent ALT, rather than toward global recombination suppression [227].

The POT1–TPP1 heterodimer coats the displaced single-stranded G-overhang within the D-loop, competing with RPA and thereby preventing activation of ATR-mediated DNA damage signaling at chromosome ends [228, 229]. Single-molecule imaging studies have revealed that TRF2–RAP1 subcomplexes exhibit markedly more dynamic binding behavior than TRF1–TIN2–TPP1–POT1 assemblies, consistent with TRF2’s role in architectural remodeling rather than static chromatin organization [230]. During S phase, TRF2 specifically recruits RTEL1 to T-loops in an S-phase-dependent manner; failure of this recruitment leads to telomere fragility phenotypes, demonstrating that T-loop resolution is a regulated, actively licensed event rather than spontaneous unwinding [231]. How aging-related changes in shelterin stoichiometry or post-translational modification alter T-loop stability in a cell-type-specific manner remains poorly characterized [232, 233]. This gap is of considerable relevance to understanding telomere biology in aging tissues.

##### Mitochondria-specific regulatory factors

Mitochondrial D-loop stability is governed by a specialized machinery operating within the highly oxidative, protein-dense environment of the mitochondrial matrix. POLγ (POLγA catalytic subunit with two POLγB accessory subunits) is the sole replicase responsible for 7 S DNA synthesis; the POLγB subunits confer structure-specific DNA binding critical for processivity and template recognition at the primed L-strand [234, 235]. A breakthrough pharmacological advance identified PZL-A, a first-in-class small-molecule allosteric activator binding at the POLγA–POLγB interface, which can restore wild-type-like processivity to disease-associated POLγ mutants in vitro and activate mtDNA synthesis in cells from patients with POLG disease [236]. This not only opens therapeutic avenues but also provides compelling evidence that the POLγA–POLγB interface is a critical regulatory hotspot that modulates D-loop formation efficiency.

TWINKLE helicase loads reversibly at the core TAS, and its loading state constitutes a molecular switch that determines whether replication terminates to form a D-loop or proceeds to produce full-length mtDNA [237]. ATAD3, an inner mitochondrial membrane-anchored ATPase, has been implicated in tethering the D-loop region to the inner membrane, potentially coupling D-loop dynamics to nucleoid organization and steroidogenesis—raising the possibility that mitochondrial D-loop formation is linked to metabolic state [238]. However, the mechanistic basis of ATAD3-mediated D-loop tethering and its relationship to metabolic signaling remain incompletely defined. Mutations in POLG, TWINKLE, and TAS cis-elements disrupt the synthesis-degradation equilibrium of 7 S DNA, leading to mtDNA depletion, multiple deletions, or tissue-specific mitochondrial disorders [239, 240]. The observation that the same D-loop regulatory axis can produce qualitatively different disease phenotypes depending on mutation type and tissue context underscores the importance of cellular context in determining outcomes. This principle applies across all three classes of D-loop but is most directly demonstrated in mitochondrial disease genetics.

##### Cell cycle coordination and post-translational modifications

HR-mediated D-loop formation is strictly confined to S and G2 phases due to the dual requirement for sister chromatid availability as a repair template and CDK-dependent phosphorylation of critical factors. CDK1/2 phosphorylation of CtIP at Ser327 and Thr847 promotes DNA end resection—the committed step toward HR [241]. In G1, 53BP1 is recruited to DSBs via RIF1 and PTIP, imposing a protective block on resection and enforcing non-homologous end joining (NHEJ). BRCA1 specifically antagonizes this 53BP1-mediated barrier in S/G2 by restraining ATM-mediated 53BP1 phosphorylation, thereby tilting the pathway balance toward HR [242].

Beyond CDK-mediated phosphorylation, D-loop regulation is pervasively controlled by post-translational modifications (PTMs). SUMOylation of RAD51 and RPA modulates their dynamics at DSBs [243]. Ubiquitination of PCNA by the RAD6–RAD18 complex creates a platform for translesion synthesis polymerases that compete with RAD51 loading [244, 245]. Phosphorylation of RAD54 regulates its translocation velocity and DNA-binding strength, affecting the balance between crossover and non-crossover outcomes [246]. While individual PTMs on specific substrates have been characterized, how the cell integrates these modifications into a coherent regulatory program remains poorly understood. This is particularly true under conditions of extensive DNA damage or replication stress. Systematic phosphoproteomic and ubiquitinomic profiling of D-loop-associated proteins under defined genotoxic conditions represents an important future direction.

### Biological function

#### Homologous recombination repair and genome stability

As a central intermediate of HR repair, the displacement loop (D-loop) plays a pivotal role in safeguarding genome stability. Following RAD51-mediated strand invasion into homologous dsDNA (see Sect.  3.2.2), the resulting D-loop intermediate is dynamically regulated to deliver high-fidelity repair outcomes. The functional emphasis below therefore focuses on how the D-loop intermediate, once formed, is dynamically regulated to deliver high-fidelity repair outcomes.

D-loop formation and dissociation are subject to exquisitely tuned dynamic regulation, and this reversibility is essential for maintaining the high fidelity of HR. The cooperative action of the anti-recombinase machinery (Sgs1–Top3–Rmi1/BLM–TOP3α–RMI1, Srs2/PARI, Mph1/FANCM) together with the recently described transcription-coupled D-loop suppression at the donor locus, all discussed in detail in Sect.  3.2.2, collectively restrain ectopic recombination and multi-invasion–driven chromosomal rearrangements, while still permitting allelic homologous repair [222, 223].

BRCA2 is also indispensable for maintaining genome stability through D-loop-associated processes. Structurally, the BRCA2 C-terminal domain TR2 interface unexpectedly induces a unique ATP–RAD51 dimer conformation that accommodates nucleation onto double-stranded B-form DNA, providing a mechanistic basis for BRCA2’s role in replication fork protection independent of canonical HR initiation [247]. Moreover, BRCA2 contributes to R-loop resolution through interaction with PCID2, a subunit of the TREX-2 complex, thereby suppressing RNA: DNA hybrid accumulation at actively transcribed loci [248]. BRCA2 deficiency leads to R-loop accumulation at promoter-proximal pause sites during transcription elongation, which has been mechanistically traced to impaired BRCA2-dependent recruitment of PAF1 to RNA polymerase II, constituting a major source of replication stress and cancer-associated genome instability [249]. In *Saccharomyces cerevisiae*, single-molecule imaging revealed that the Rad51 paralog complex Rad55–Rad57 functions as a molecular chaperone that promotes assembly of the Rad51 recombinase filament through transient interactions, providing a paradigm for paralog-mediated mediator activity [250]. This chaperone-like function is conserved in mammals: the five human RAD51 paralogs (RAD51B, RAD51C, RAD51D, XRCC2 and XRCC3) assemble into the BCDX2 (RAD51B–RAD51C–RAD51D–XRCC2) and CX3 (RAD51C–XRCC3) complexes, which stimulate nucleation and extension of RAD51 nucleoprotein filaments to safeguard genome integrity [251]. Pathogenic germline variants in these human paralogs disrupt RAD51 filament dynamics and predispose carriers to breast, ovarian and other cancers as well as Fanconi anemia–like disorders [252].

#### Telomere maintenance and chromosome end protection

As described in Sect.  3.1 and 3.2.2, the telomeric D-loop embedded within the T-loop architecture provides essential chromosome end protection through the combined action of TRF2-mediated strand invasion and shelterin-dependent stabilization. Here we focus on the dynamic regulation and functional consequences of telomeric D-loops in telomere length homeostasis, replicative senescence, and the ALT pathway.

Because telomeric D-loops must be maintained throughout interphase yet resolved during S phase to allow replication fork passage, their dynamics are subject to precise cell-cycle-dependent control. The CDK phosphatase PP6R3 dephosphorylates TRF2 at Ser365 specifically during S phase, enabling TRF2 to recruit the RTEL1 helicase through an interaction mediated by the C4C4 metal-coordinating motif of RTEL1 and the TRFH domain of TRF2 [231]. This creates a narrow temporal window during which RTEL1 directly unwinds the D-loop embedded within the T-loop, facilitating replisome passage. Re-phosphorylation of TRF2-Ser365 by CDKs outside of S phase releases RTEL1 from telomeres, thereby protecting T-loops from promiscuous unwinding and inappropriate ATM activation. Failure of this regulated D-loop dissolution, as exemplified by the Hoyeraal–Hreidarsson syndrome mutation RTEL1R1264H which disrupts the C4C4 motif and abolishes the TRF2–RTEL1 interaction, leads to catastrophic processing of persistent T-loops by SLX4-associated nucleases. This generates extrachromosomal telomere circles and rapid telomere shortening associated with the severe clinical phenotype of this telomere biology disorder [231].

The dynamic balance between T-loop formation and resolution has significant implications for telomere length homeostasis and replicative senescence. In telomerase-expressing cells, such as germline cells, embryonic and adult stem cells, and the majority of cancer cells that reactivate telomerase to achieve replicative immortality [253, 254]. When RTEL1 unwinds the telomeric D-loop in S phase, the liberated 3′ overhang becomes accessible to telomerase for elongation, coupling T-loop resolution with telomere extension. Conversely, stabilization of the D-loop by TRF2 restricts telomerase access and limits extension outside of S phase, constituting a negative-feedback mechanism that prevents excessive telomere elongation. In ALT-positive cancer cells, which maintain telomere length through HR-dependent mechanisms rather than telomerase and account for approximately 10–15% of all cancers [255], the telomeric D-loop serves as a critical recombination intermediate. ALT is not restricted to a single recombination route but encompasses multiple, partially overlapping HR-based pathways, including inter-telomeric homologous recombination and telomere sister-chromatid exchange (T-SCE), conservative break-induced replication triggered by replication fork collapse, and RAD52-dependent mitotic DNA synthesis (MiDAS) that completes under-replicated telomeres in G2/M [256–258]. These processes converge on D-loop intermediates as common platforms for homology-directed telomere extension. Following replication fork collapse at telomeres, RAD51- and RAD52-dependent strand invasion into a homologous telomeric template generates D-loops that serve as platforms for BIR-mediated DNA synthesis [258, 259]. Mechanistically, RAD52 drives D-loop formation on telomeric ssDNA coated by RPA and promotes BIR-coupled DNA synthesis in G2/M, whereas RAD51 primarily suppresses telomere fragility by protecting stalled replication forks rather than directly catalyzing strand invasion at ALT telomeres.

The fate of telomeric D-loops in ALT cells determines whether recombination leads to productive telomere elongation or deleterious chromosomal rearrangements. Extended D-loops can be resolved by two competing machineries: the BTR complex (BLM–TOP3α–RMI1/RMI2) dissolves D-loops to generate non-crossover elongation products, while the SMX complex (SLX4–SLX1–MUS81–EME1–XPF–ERCC1) cleaves D-loops to produce telomere sister-chromatid exchanges (T-SCEs) and crossovers [260]. The antagonism between these two D-loop-processing complexes constitutes a molecular switch that determines the balance between productive telomere elongation and potentially pathological recombination events in ALT cells. Notably, FANCM regulates BTR complex activity at ALT telomeres through its translocase activity; FANCM loss in ALT-positive cells markedly elevates D-loop-dependent telomeric DNA synthesis and exacerbates ALT phenotypes, revealing FANCM as a candidate therapeutic vulnerability in ALT-driven tumors [261].

#### Cancer development and therapeutic implications

The central role of D-loops in HR-mediated DSB repair positions the D-loop regulatory axis as a pivotal determinant of cancer susceptibility, tumor progression, and therapeutic response. Mutations in core D-loop effectors, most prominently BRCA1 and BRCA2, confer homologous recombination deficiency (HRD). HRD is characterized by impaired D-loop formation, aberrant replication fork dynamics, and consequent genome instability that drives tumorigenesis [247, 262]. Pathogenic variants in BRCA1/2 dramatically increase lifetime risk of breast and ovarian cancer in carriers. The BRCAness spectrum, encompassing mutations in parallel or upstream HR pathway genes including PALB2, ATM, CHEK2, RAD51C, RAD51D, and BARD1, expands the potentially HRD-attributable tumor population substantially [262]. In high-grade serous ovarian cancer, approximately 50% of cases carry HRD-associated alterations, with significant prevalences also reported in triple-negative breast cancer, metastatic prostate cancer, and pancreatic ductal adenocarcinoma [263].

The mechanistic link between D-loop dysfunction and cancer therapy is most directly illustrated by the synthetic lethality of PARP inhibitors (PARPi) in HRD tumors. In HR-proficient cells, PARP1 functions primarily as a sensor of DNA single-strand breaks (SSBs) and other DNA lesions through its N-terminal zinc-finger domains, where it catalyzes poly(ADP-ribosyl)ation to signal damage and recruit downstream repair factors such as XRCC1, thereby promoting SSB repair largely outside the context of ongoing replication [264, 265]. PARPi both block PARP1 catalytic activity and trap the enzyme on DNA repair intermediates; the trapped complexes obstruct progressing replication forks during S phase, leading to fork collapse and the formation of replication-associated DSBs [266, 267]. In HRD cells lacking functional D-loop-mediated HR, these DSBs accumulate and trigger genomic catastrophe [268]. Multiple FDA-approved PARPi, including olaparib, niraparib, rucaparib, and talazoparib, are now standard-of-care treatments for BRCA-mutated ovarian, breast, pancreatic, and prostate cancers [263, 269]. The relationship between RAD51-mediated D-loop formation and PARPi sensitivity is underscored by functional assays: immunofluorescence quantification of nuclear RAD51 foci, a direct readout of productive D-loop formation capacity, has emerged as a robust biomarker for predicting PARPi response across multiple tumor types beyond BRCA-mutated cancers [270, 271].

Despite initial clinical efficacy, acquired PARPi resistance represents a critical oncological challenge and is mechanistically linked to restoration of D-loop formation capacity. The most common resistance mechanism is the acquisition of secondary (reversion) mutations in BRCA1/2, RAD51C, RAD51D, or PALB2 that re-establish reading frame and restore functional HR [263]. Additional resistance mechanisms include: (i) upregulation of replication fork protection factors (53BP1 loss in BRCA1-deficient cells restores end resection and partial HR); (ii) reduced PARP trapping through downregulation of PARP1 expression or mutations in its DNA-binding domain; (iii) enhanced drug efflux via ABC transporters; and (iv) restoration of RAD51 loading at stalled forks through loss of antagonizing factors such as RADX [263, 272]. To overcome resistance, combinatorial strategies including co-targeting the PI3K/AKT/mTOR, ATR/CHK1, or WEE1 axes alongside PARPi are under active clinical evaluation, with the goal of maintaining or re-inducing HRD [263].

A clinically significant emerging vulnerability involves direct disruption of the RAD51–BRCA2 protein–protein interaction required for D-loop initiation. Small-molecule inhibitors of the BRCA2 BRC repeat–RAD51 interface, exemplified by compound RS-35d and its pharmacologically optimized enantiomers, selectively impair HR in BRCA2-proficient cells. These inhibitors demonstrate synergistic synthetic lethality with olaparib in BRCA2-wild-type pancreatic cancer models. This conceptually extends the PARPi therapeutic window beyond genetically HRD tumors [273]. More broadly, overexpression of RAD51 is observed across multiple solid tumor types and correlates with resistance to DNA-damaging therapies; pharmacological RAD51 inhibition or its depletion re-sensitizes resistant tumors to PARPi and platinum-based agents, further validating D-loop formation capacity as a druggable oncological target [273].

#### Meiotic recombination and the generation of genetic diversity

In the context of meiosis, D-loops are indispensable intermediates that couple programmed DSB repair to homologous chromosome pairing, synapsis, and crossover formation, processes that are fundamental to genetic diversity and faithful chromosome segregation. Meiotic recombination is initiated when the meiosis-specific topoisomerase-like enzyme SPO11 catalyzes programmed DSBs at preferred genomic sites (hotspots), which in many vertebrates are specified by the PRDM9 histone methyltransferase binding in a sequence-specific manner [274]. Following a two-step 5′-to-3′ resection cascade in which the MRN complex (MRE11–RAD50–NBS1) together with CtIP performs short-range processing and removes SPO11–oligonucleotide complexes, while the long-range tracts are extended by EXO1, with auxiliary contributions from the BLM helicase and DNA2 nuclease [275, 276], the resulting 3′ ssDNA overhangs are coated first by RPA and subsequently by co-loaded recombinases RAD51 and the meiosis-specific paralogue DMC1, which form right-handed helical presynaptic filaments that mediate homology search and strand invasion to generate D-loop intermediates between the broken chromosome and the homologous chromosome template [274, 277].

A defining mechanistic feature of meiotic D-loop regulation is interhomolog (IH) bias. This is the preferential use of the homologous chromosome rather than the sister chromatid as the repair template. IH bias is essential for productive crossover formation and homolog recognition. This bias is achieved through a functional division of labor between RAD51 and DMC1: RAD51 assists presynaptic filament assembly but its strand exchange activity is suppressed by the inhibitory factor Hed1 (TRIP13 pathway in mammals), reserving productive strand invasion for DMC1 [278]. This functional distinction has a structural basis: D-loops formed by DMC1 are substantially more resistant to dissociation by branch-migration proteins such as RAD54 and BLM than those formed by RAD51, providing a molecular explanation for why DMC1-mediated intermediates are preferentially channeled toward stable interhomolog crossovers rather than dissolved non-crossovers [279].

**Fig. 7:**
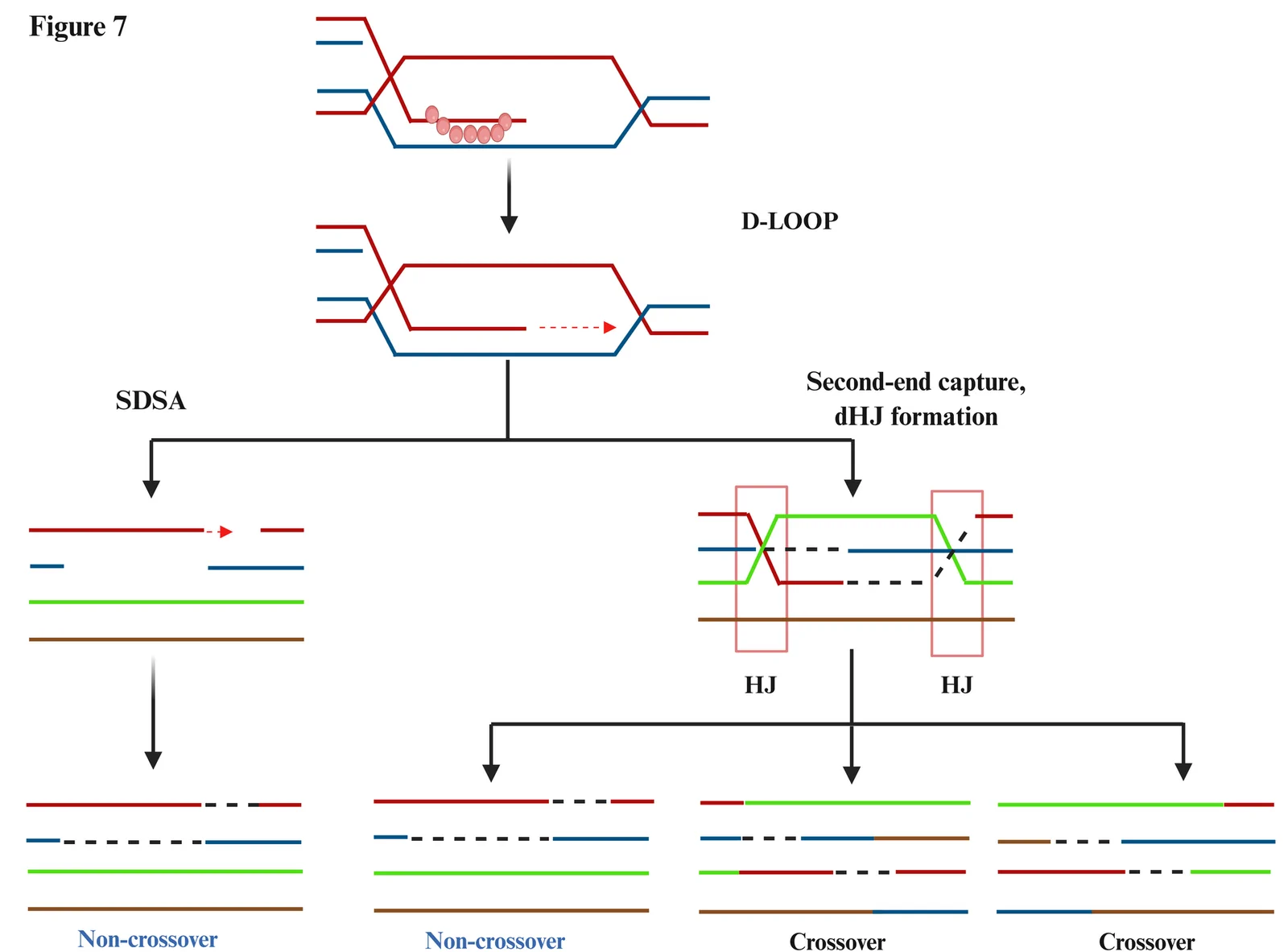
Downstream processing of D-loop intermediates during homologous recombination (HR). Following strand invasion and D-loop formation, the invading 3′ end is extended by DNA synthesis (dashed arrow) [24]. The D-loop intermediate can then be channeled into two major pathways. In the Synthesis-Dependent Strand Annealing (SDSA) pathway (left), the newly synthesized strand is displaced and re-anneals with the resected ssDNA on the opposite end, yielding a non-crossover (NCO) product. Alternatively, second-end capture of the displaced strand stabilizes the D-loop and promotes the formation of a double Holliday Junction (dHJ) intermediate (right) [284]. The dHJ can be resolved by either non-crossover-biased dissolution or by endonucleolytic resolution, generating either NCO or crossover (CO) products depending on the orientation of cleavage. Red and blue lines represent the two parental DNA duplexes; green lines denote the invading or newly synthesized strand; dashed lines indicate newly synthesized DNA. Colored boxes highlight Holliday junction branch points

The fate of nascent meiotic D-loops is a key determinant of crossover versus non-crossover outcome, with profound consequences for genome evolution and fertility. A subset of D-loops are stabilized by the ZMM (Zip1–Zip2–Zip3–Zip4–Msh4–Msh5–Spo16) family of proteins, which protect D-loops from premature dissolution by helicases such as FANCM/Fml1 and Sgs1/BLM, and channel them through the double Holliday junction (dHJ) pathway toward obligate Class I crossovers that are subject to interference [280]. Class I crossovers constitute the majority of meiotic crossovers in most organisms and their even spacing along chromosomes—enforced by crossover interference—guarantees that each homolog pair receives at least one obligate crossover necessary for proper meiosis I segregation [281]. The remaining DSBs are resolved as non-crossovers through synthesis-dependent strand annealing (SDSA), in which D-loops are unwound after limited DNA synthesis by dedicated helicases including the FANCM/Fml1–MHF complex and Sgs1/BLM, and the extended 3′ end re-anneals with the second resected end without forming a dHJ [282, 283] (Fig. 7).

A homeostatic mechanism ensures that a minimum number of crossovers are formed regardless of variation in DSB numbers: when global DSB levels are reduced, the crossover/non-crossover ratio increases compensatorily. Callender and Hollingsworth demonstrated that this homeostasis operates through the RAD51/DMC1-mediated interhomolog bias system and requires functional Dmc1; in *dmc1 hed1* cells where Rad51 acts in place of Dmc1, homeostatic compensation is abolished, because the crossover/non-crossover ratio is already maximally elevated and cannot be further increased [285]. Genome-wide ChIP-seq mapping of RAD51, DMC1, and RPA in mouse meiosis has provided spatial resolution of recombination intermediates: RAD51 occupies twin peaks flanking hotspot centers corresponding to the resected DSB ends, while DMC1 has a broader footprint at hotspot centers consistent with its role in interhomolog D-loop formation [286].

Perturbation of D-loop regulatory factors in the germline has severe reproductive consequences that illuminate the essential nature of this pathway. Loss of the FIRRM/C1orf112 protein, which forms a complex with the FIGNL1 AAA-ATPase to promote RAD51 and DMC1 disassembly from chromatin, results in persistent presynaptic filaments. These filaments accumulate on chromatin even in the absence of SPO11-generated DSBs. This loss impairs ZMM protein (MSH4, TEX11) focus formation by approximately 2.5-fold and causes meiotic arrest in pachytene. These defects ultimately result in complete male infertility in mice [287, 288]. The finding that RAD51 and DMC1 accumulate in FIRRM-depleted spermatocytes independently of SPO11-catalyzed DSBs underscores the importance of active filament disassembly in channeling recombination intermediates through controlled D-loop formation rather than aberrant filament propagation. Collectively, these findings establish meiotic D-loops as indispensable molecular nodes linking programmed DNA damage to the creation of genetic diversity and the fidelity of reproductive cell division.

#### Mitochondrial diseases and the mtDNA D-loop region

The mtDNA D-loop region, whose structural organization and regulatory elements (OH, HSP, LSP) have been described in Sect.  3.1, serves as the major non-coding regulatory hub of the mitochondrial genome. Its exceptionally high mutation rate (5–10× above mtDNA coding regions) reflects the oxidative environment and limited DNA repair capacity of the mitochondrial matrix [152]. Below we focus on the pathological consequences of D-loop perturbation in mitochondrial disorders, neurodegeneration, and cancer.

Mutations within the D-loop region not only affect the replication efficiency and transcriptional levels of mtDNA but also contribute to diverse disease phenotypes through resultant mitochondrial dysfunction. Mutations in *POLG* (encoding POLγ) and *TWNK* (encoding the TWINKLE helicase), together with structural variants within the TAS cis-element that governs 7 S DNA turnover, disrupt the regulated equilibrium of mtDNA D-loop maintenance, leading to mtDNA depletion syndromes or multiple large-scale deletions associated with a wide spectrum of clinical manifestations ranging from progressive external ophthalmoplegia to Alpers–Huttenlocher syndrome [289].

In neurodegenerative diseases, abnormal epigenetic modifications of the D-loop region have emerged as a key research focus. A study involving patients with amyotrophic lateral sclerosis revealed a significant decrease in D-loop methylation levels in peripheral blood lymphocytes from sporadic ALS patients, which was negatively correlated with mtDNA copy number, potentially representing a compensatory mechanism against mitochondrial dysfunction. Characteristic differences across ALS subtypes were identified: *SOD1* mutation carriers exhibited the lowest methylation levels, sporadic patients showed intermediate levels, while *C9orf72* mutation carriers displayed relatively higher levels, suggesting disease subtype as a primary determinant of epigenetic status at the D-loop region [290]. Furthermore, droplet digital PCR (ddPCR)-based quantification of mtDNA D-loop copy number variation has been proposed as a non-invasive biomarker of mitochondrial dysfunction in aging, metabolic syndrome, and multiple cancers, though interpretation requires caution because anomalous amplification characteristics of the D-loop region can confound accurate mtDNA copy number estimation compared to coding regions [291].

### Detection methods

Accurate detection of D-loops is essential for elucidating their roles in homologous recombination, DNA repair, and mitochondrial genome biology. D-loop detection methods can be broadly categorized based on their application contexts and technical approaches, encompassing both classical biochemical techniques and cutting-edge single-molecule methodologies. For homologous recombination-mediated D-loops, the gel-based D-loop assay represents the most classical and widely utilized approach. This method typically employs Rad51-mediated strand invasion reactions where a 5′-radiolabeled single-stranded DNA oligonucleotide invades a homologous supercoiled double-stranded DNA template, with the resulting D-loop products separated by native agarose or polyacrylamide gel electrophoresis and detected via autoradiography [292]. As a non-radioactive alternative, the radiolabel on the invading ssDNA can be substituted with a covalently attached fluorophore such as fluorescein (FAM), Cy5, or Alexa Fluor 488, allowing direct fluorescence imaging of D-loop products [293]. This fluorophore-labeling strategy has been further extended to homogeneous, microplate-based formats, in which a quencher-labeled ssDNA pairs with a fluorophore-labeled duplex so that strand invasion produces a real-time change in fluorescence intensity, providing a sensitive and high-throughput-compatible readout for mechanistic and inhibitor-screening studies [294, 295]. Alternative detection strategies using fluorescent dyes such as DAPI (4′,6-diamidino-2-phenylindole) or ethidium bromide offer radiation-free alternatives, though with slightly reduced sensitivity compared to radioactive labeling [292]. A notable methodological advance emerged in 2020 with the development of bisulfite treatment coupled with single-molecule real-time sequencing, which enables high-resolution characterization of D-loop structures at near base-pair resolution. Shah et al. developed an in vitro approach employing non-denaturing bisulfite treatment to selectively modify cytosines on displaced single-stranded DNA within D-loops to uracil, creating permanent molecular signatures that permit precise determination of individual D-loop length, position, and distribution. These capabilities were previously unattainable with traditional restriction-digestion methods [296]. Complementing these in vitro techniques, proximity ligation-based assays such as the D-loop capture (DLC) and D-loop extension (DLE) methods have substantially improved in vivo detection and quantification of D-loop dynamics in *Saccharomyces cerevisiae* [297–299]. These approaches utilize psoralen crosslinking to preserve heteroduplex DNA within D-loops, followed by restriction digestion and intramolecular ligation under dilute conditions, enabling sensitive quantitative PCR-based detection with a dynamic range exceeding three orders of magnitude [297, 298]. Recent methodological refinements incorporating psoralen de-crosslinking prior to qPCR now permit absolute quantification of D-loop joint molecules in cell populations and have proven instrumental in dissecting the dynamic processing of D-loops by recombination regulators including Srs2, Mph1, and the Sgs1-Top3-Rmi1 helicase-topoisomerase complex [297, 299]. For structural visualization, electron microscopy (EM) enables direct observation of D-loop intermediates, providing invaluable morphological information on synaptic contacts between invading and template DNA molecules, though this technique requires specialized sample preparation and expertise [300]. Additionally, innovative applications of two-dimensional agarose gel electrophoresis followed by in-gel hybridization have been successfully adapted from telomeric t-loop detection methodologies, exploiting the structural similarity between D-loops and rolling circle replication intermediates to achieve specific detection based on characteristic sigmoidal migration patterns [301, 302]. The integration of these complementary methodological platforms, spanning from single-molecule resolution bisulfite sequencing and proximity ligation-based quantitative assays to electron microscopy and advanced PCR techniques, continues to refine our mechanistic understanding of D-loop formation, processing, and biological functions across diverse cellular contexts.

## Comparative overview

R-loops and D-loops are two architecturally related yet mechanistically distinct three-stranded nucleic acid structures whose divergent origins fundamentally determine their biological deployment (Table 1). R-loop formation is primarily governed by thermodynamic determinism: RNA: DNA hybrid duplexes adopt an intermediate helical geometry between canonical A-form and B-form DNA, a conformation of lower free energy than the B-form duplex it displaces, thereby conferring on nascent RNA transcripts an intrinsic propensity to invade the DNA template strand. This thermodynamic favorability renders R-loops a pervasive and persistent genomic liability during transcription, necessitating continuous surveillance by a dedicated resolution machinery comprising RNA: DNA helicases (notably SETX, DHX9, and DDX5) and ribonucleases (RNase H1 and H2) [30, 32, 37]. In contrast, DNA: DNA heteroduplexes lack the thermodynamic advantage intrinsic to RNA: DNA hybrids, so D-loops generally do not arise spontaneously and instead depend on context-specific factors that overcome the energetic barrier to strand invasion. Importantly, the molecular routes to D-loop assembly differ substantially among subtypes. Recombination D-loops formed during homologous recombination repair are catalyzed by ATP-dependent recombinases, principally RAD51 in somatic cells and DMC1 during meiosis, which assemble on resected 3′ single-stranded DNA tails to drive homology search and strand exchange [184, 205]. Telomeric D-loops embedded within the t-loop configuration are instead nucleated by the shelterin component TRF2, which bends and wraps duplex telomeric DNA to facilitate invasion of the 3′ G-overhang; recent in vivo evidence indicates that this process can proceed independently of RAD51, distinguishing t-loop assembly from canonical recombinase-driven invasion [163, 303]. Mitochondrial D-loops follow yet another route, arising as a byproduct of strand-displacement replication: an LSP-derived RNA primer first establishes an R-loop at the leading-strand origin, POLγ then extends nascent heavy-strand DNA that is prematurely terminated at the termination-associated sequences, and the resulting 7 S DNA remains hybridized to the parental light strand to maintain the triple-stranded structure [304, 305]. Thus, although all D-loops require facilitating factors rather than spontaneous nucleation, the responsible machinery is subtype-specific, encompassing ATP-driven recombinases, shelterin-mediated topological remodeling, and transcription-coupled replication initiation. Taken together, R-loops represent spontaneous biophysical traps that are actively resolved to maintain genomic integrity, whereas D-loops are highly coordinated, transient intermediates whose formation is licensed only at defined cell cycle stages (S and G2 phases) or specific chromosomal contexts (e.g., telomeres, mitochondrial non-coding regions) to enable targeted repair, replication, or end protection [5, 28, 185].

**Table 1:** Key regulatory factors of R-loops and D-loops

Functional Category	Factor	Primary Role
R-loop Formation Promoters	G-rich sequences [62], GC skew [30, 37]	Provide intrinsic sequence determinants for R-loop formation
	Negative supercoiling [68, 69]	Lowers the energy barrier for strand separation, facilitating RNA invasion
R-loop Resolvases	SETX [40], DHX9 [41], DDX5 [70]	RNA: DNA helicases that unwind the hybrid
	RNase H1, RNase H2 [3, 40]	Ribonucleases that degrade the RNA moiety of the hybrid
R-loop Epigenetic	METTL3, YTHDC1, YTHDF2 [79, 80]	Mediate m6A modification and recognition, influencing R-loop stability and outcome
	TUG1 (lncRNA), LSD1 [83, 113]	Non-coding RNA and chromatin modifier that regulate R-loop dynamics
D-loop Recombinases	RAD51, DMC1 [14, 205, 278]	Catalyze strand invasion to form the core D-loop structure
D-loop Promoters/Stabilizers	BRCA2, PALB2 [206, 212, 247]	Mediate RAD51 loading and filament stabilization
	RAD54 [214–216]	Promotes strand invasion and heteroduplex extension; remodels RAD51
	RAD51AP1 [106]	Enhances RAD51-mediated D-loop formation, notably at telomeres
D-loop Dissolvases/Anti-recombinases	BLM-TOP3α-RMI1 [220]	Dissolves D-loops to prevent crossover formation
	RTEL1 [169, 221]	Unwinds HR D-loops; resolves telomeric D-loops during S phase
	Srs2, FANCM [219, 222, 223]	Disrupts RAD51 filaments or D-loop intermediates
D-loop Telomere-specific Regulators	TRF2 [167, 226, 306]	Architectural shelterin protein that facilitates and stabilizes the telomeric D-loop
	POT1-TPP1 [231, 307]	Sequesters the displaced G-rich ssDNA within the telomeric D-loop, preventing ATR activation
D-loop Mitochondrial Regulators	POLγ (POLγA-POLγB) [234–236]	mtDNA polymerase responsible for 7 S DNA synthesis within the D-loop
	TWINKLE [237, 239]	Mitochondrial helicase that regulates 7 S DNA synthesis and D-loop turnover
	ATAD3 [238]	Inner membrane ATPase implicated in tethering the D-loop region

*Abbreviations: HR *homologous recombination, *ALT *alternative lengthening of telomeres, *mtDNA *mitochondrial DNA, *ssDNA *single-stranded DNA, *m6A *N6-methyladenosine

A further determinant distinguishing R-loop and D-loop biology is the fidelity of Watson-Crick base pairing, with mismatches exerting strikingly different effects on each structure. For R-loops, single internal mismatches between the nascent transcript and template DNA significantly lower hybrid melting temperature and shorten hybrid lifetime, providing a passive thermodynamic filter that biases R-loop formation toward loci of high RNA template complementarity [30, 37]. Mismatches can also distort the A-form-like geometry required for RNase H1 and H2 recognition, thereby coupling base-pairing fidelity to R-loop turnover kinetics. For D-loops, fidelity is enforced through both biophysical and enzymatic checkpoints. RAD51- and RecA-mediated strand exchange tolerates moderate sequence divergence, with studies in budding yeast showing efficient break-induced replication when mismatches are placed every eighth to tenth base pair, although stable strand invasion still requires approximately six to eight contiguous paired bases [308]. Once heteroduplex DNA is formed, the resulting mismatches are recognized by the mismatch repair machinery (MSH2-MSH6/MSH3 in eukaryotes; MutS-MutL in bacteria), which either consolidates the heteroduplex through gene conversion or triggers heteroduplex rejection via the Sgs1/BLM helicase to dismantle homeologous joint molecules [309–311]. Thus, base-pairing fidelity is filtered passively for R-loops through hybrid thermodynamics but actively for D-loops through MMR-dependent quality control.

Despite these divergent origins, both structures fulfill indispensable physiological roles that collectively safeguard genome integrity and support fundamental cellular programs. R-loops contribute to transcription termination, gene expression regulation, the DNA damage response, chromatin remodeling at CpG island promoters, and immunoglobulin class-switch recombination [3–5, 93, 94]. D-loops, in turn, mediate high-fidelity HR-based DSB repair, provide structural chromosome end protection within the T-loop architecture at telomeres, and drive mtDNA synthesis through the strand-displacement replication mechanism [145, 149, 151]. Critically, however, dysregulation of either structure undermines the very genomic integrity it normally protects. Aberrant accumulation or impaired clearance of R-loops engenders replication fork stalling, transcription–replication conflicts, and exposure of the displaced non-template strand to endogenous mutagens [43, 44]. Conversely, imbalances in D-loop formation and dissolution can precipitate ectopic recombination, chromosomal rearrangements through multi-invasion mechanisms, or replication fork collapse, collectively amplifying the burden of DNA damage and structural variation.

A cardinal feature shared by both structures is the generation of a displaced single-stranded DNA (ssDNA) loop, which represents a region of acute topological vulnerability. The manner in which cells manage this ssDNA, however, diverges sharply and constitutes a fundamental determinant of biological outcome. Within R-loops, the displaced non-template strand is bound by RPA, which colocalizes with R-loops in cells. Rather than acting as a stabilizing chaperone, RPA here serves as a sensor that recruits and stimulates RNase H1 to drive RNA: DNA hybrid resolution [124]. Critically, RPA engagement does not preclude secondary-structure formation: at G-rich loci, the displaced strand can still fold into G-quadruplex (G4) structures, yielding a mutually reinforcing G-loop architecture that escapes timely resolution and amplifies genomic instability [59, 64]. G4 stabilization of the displaced ssDNA inhibits re-annealing with the template strand, prolonging R-loop lifetime and rendering the ssDNA accessible to endogenous nucleases and cytidine deaminases, including APOBEC family enzymes [312]. In contrast, the displaced ssDNA within D-loops is subject to active and context-specific sequestration. During HR-mediated DSB repair, the ssDNA overhangs are dynamically coated by Replication Protein A (RPA), which prevents secondary structure formation and nucleolytic attack while serving as a platform for recombinase loading [177, 178]. Within telomeric T-loops, the displaced G-rich overhang is specifically sequestered by the POT1–TPP1 heterodimer, preventing activation of ATR kinase-dependent damage signaling at chromosome termini [167, 168]. Thus, the divergent outcomes of R-loops and D-loops are dictated not by the presence of ssDNA-binding factors per se, but by the functional context of their engagement: RPA at R-loops is coupled to resolution machinery and frequently outcompeted by G4 folding, whereas at D-loops, stringent ssDNA protection is a prerequisite for their function as stable, productive intermediates.

Beyond protein-mediated protection, the displaced ssDNA in both R-loops and D-loops is subject to mutagenic attack that constitutes a significant source of somatic mutations in cancer. In R-loops, the exposed non-template strand is a preferred substrate for APOBEC3A and APOBEC3B cytidine deaminases, which catalyze C-to-U conversions that are subsequently processed into C-to-T transition mutations; genome-wide analyses have demonstrated that APOBEC-signature mutations are significantly enriched at R-loop-forming loci in multiple cancer types [313]. In D-loops, the displaced strand is similarly vulnerable: during BIR and long-tract gene conversion, the displaced strand within extended D-loops is exposed for prolonged periods, providing a window for spontaneous deamination and oxidative damage that contributes to the elevated mutagenesis observed at BIR-repaired loci [314]. The convergent mutagenic vulnerability of displaced ssDNA in both structures raises the possibility that common protective mechanisms exist; indeed, recent work has implicated ssDNA-binding proteins RPA and RAD52 in minimizing damage to exposed strands in both contexts, although the degree of mechanistic overlap remains to be established [124].

Although R-loops and D-loops are conventionally studied as distinct entities within transcription and DNA repair biology, respectively, they converge functionally at multiple genomic loci and constitute components of a continuous regulatory axis in genome homeostasis. The most prominent point of intersection is the transcription–replication conflict [44, 115]. When an advancing replication fork encounters a persistent R-loop in either co-directional or counter-directional orientation, the resulting replication stress and fork stalling can generate one-ended or canonical DSBs. Resolution of such R-loop-derived lesions may require D-loop formation via homologous recombination to restart the collapsed fork, thereby establishing a mechanistic link in which the pathological output of one structure (the R-loop) creates the substrate that necessitates the formation of the other (the D-loop) [49, 101]. This coupling is reinforced by shared regulatory components. Helicases such as FANCM and SETX participate in R-loop resolution at transcription–replication conflict sites while simultaneously influencing the dynamics of recombination intermediates at stalled forks [74, 75].

A key regulatory parallel between R-loops and D-loops is their distinct but complementary cell cycle dependencies. R-loops form throughout the cell cycle as an intrinsic consequence of transcription, with their steady-state levels modulated by the availability of resolving enzymes and RNA processing factors rather than by direct cell cycle gating [28]. In S phase, R-loop accumulation at active genes poses an acute threat to genome stability because of the increased probability of transcription–replication conflicts; accordingly, several R-loop resolving factors, including SETX and the FANC pathway, are transcriptionally or post-translationally upregulated during S and G2 phases to buffer this risk [315]. D-loops, by contrast, are temporally restricted by design. HR-mediated D-loops are confined to S and G2 phases through CDK-dependent phosphorylation of end-resection factors (CtIP) and the BRCA1-mediated antagonism of 53BP1 that collectively license resection and RAD51 loading [177]. Telomeric D-loops within T-loops must be maintained during interphase for end protection but actively resolved during S phase by the RTEL1 helicase to permit replication fork passage, a cycle that is regulated by TRF2-Ser365 phosphorylation [306]. Mitochondrial D-loops, uniquely, appear to be maintained continuously in a replication-coupled steady state, with 7 S DNA undergoing rapid turnover with a half-life of approximately one hour regardless of cell cycle phase [143]. This comparison reveals a regulatory gradient: from R-loops, which form constitutively and require continuous suppression, through HR D-loops, which are strictly licensed, to mitochondrial D-loops, which persist as quasi-stable replication intermediates. Understanding how these distinct temporal programs interact, particularly when cell cycle checkpoints are compromised in cancer, represents a significant unresolved question.

The functional interplay between R-loops and D-loops is particularly evident in telomere biology and ALT-driven cancers. At telomeres, TERRA-associated R-loops modulate telomeric chromatin architecture and stability, while the telomeric D-loop embedded within the T-loop provides structural chromosome end protection [103–105, 145]. In ALT-positive cells, which maintain telomere length through HR-based BIR rather than telomerase, TERRA R-loops appear to promote recombination-driven telomere elongation through a process that depends, at least in part, on the formation and maintenance of D-loop intermediates, consistent with an R-loop to D-loop switch mediated by RAD51AP1 and RAD52 [24]. At a broader level, BRCA1 functions as a critical molecular nexus connecting these two structures: it facilitates R-loop resolution through recruitment of SETX and coordination with the FANC pathway, while simultaneously promoting D-loop formation and HR pathway choice by counteracting 53BP1-mediated resection inhibition in S/G2 phase [212, 213, 242]. These examples suggest that non-canonical nucleic acid structures may be coordinated through higher-order regulatory networks in which the decisive determinants are not simply the abundance of individual structures, but rather the dynamics of switching, competition, and cooperation among distinct nucleic acid conformations, governed by the availability and activity state of shared regulatory proteins.

As noted above, R-loops and D-loops are regulated by a complex network involving multiple factors. R-loop homeostasis is shaped by a multi-layered network encompassing DNA sequence features (GC skew, G-richness), DNA topology, RNA modifications (m6A and m5C), non-coding RNA interactions, chromatin state, and epigenetic marks [40, 77, 79, 80]. These factors collectively sculpt the spatiotemporal landscape of R-loop formation, stabilization, and resolution. D-loop dynamics, by contrast, are more directly constrained by DNA superhelical tension, recombinase nucleoprotein filament assembly and disassembly kinetics, cell cycle phase, post-translational modifications of key regulators, and a network of accessory proteins that coordinate strand exchange intermediates. At the systems level, these multilayered regulatory programs reflect the delicate equilibrium that the cell must maintain between genomic stability and the controlled genomic rearrangements necessary for accurate repair, chromatin remodeling, and adaptive responses to genotoxic stress. Importantly, although R-loops are primarily embedded within transcription and RNA metabolism programs whereas D-loops are principally embedded within DNA repair and recombination circuits, the phenotypic consequences of their dysregulation frequently emerge through cross-coupling via shared molecular substrates, competition for common protein effectors, and convergence on shared stress-response pathways. This reinforces the view that these structures should be studied not in isolation, but as components of an integrated genome surveillance and maintenance network.

## Conclusions and prospects

From a translational medicine perspective, the central roles of R-loops and D-loops in diverse disease contexts create significant opportunities for therapeutic intervention and patient stratification. In oncology, targeting R-loop homeostasis has emerged as a promising therapeutic strategy. Inhibitors or modulators of R-loop processing factors, including RNase H enzymes, DHX9, and SETX, are under active investigation and may confer novel synthetic lethal vulnerabilities in HRD tumors, including BRCA1/2-deficient cancers, potentially complementing or extending the clinical utility of existing PARP inhibitor regimens [40, 41, 70, 124]. RAD52 inhibitors, which selectively impair residual recombination in BRCA2-deficient cells, have similarly been proposed as candidates for HRD-associated malignancies [316]. METTL3 inhibitors (e.g., STM2457, STC-15) represent an additional avenue by disrupting m⁶A-mediated R-loops homeostasis, triggering double-stranded RNA accumulation, activating innate immune signaling, and enhancing anti-tumor immunity, an approach that has begun to enter clinical evaluation [125, 126]. In neurodegenerative diseases, attenuation of pathological R-loop accumulation, for example in C9ORF72-associated amyotrophic lateral sclerosis and frontotemporal dementia (FTD) or in SMN1 mutation-associated spinal muscular atrophy, holds therapeutic promise by reducing cumulative DNA damage burden, suppressing neuroinflammatory signaling downstream of cGAS–STING activation, and preserving neuronal viability [129, 317]. In the context of mitochondrial disorders, small-molecule activators that restore mutant POLγ activity, exemplified by the recently described allosteric activator PZL-A which targets the POLγA–POLγB interface, represent a first-in-class pharmacological approach to rescuing mtDNA D-loop dynamics in POLG-associated disease, offering proof of principle that the mitochondrial D-loop regulatory axis is pharmacologically accessible [236].

Despite this therapeutic potential, the realization of translational applications faces substantial practical challenges that demand careful consideration. Because R-loops fulfill essential physiological functions, including transcription termination, chromatin remodeling, and the DNA damage response, broadly inhibiting their formation or non-selectively enhancing their clearance risks disrupting transcriptional homeostasis and impairing normal genome regulation, thereby constraining the viable therapeutic window [5, 28, 94]. Similarly, pharmacological manipulation of the D-loop/HR axis requires precise attention to pathway choice: interventions that globally suppress D-loop formation risk impairing genomic repair fidelity, whereas those that stabilize D-loops beyond physiological thresholds may promote ectopic recombination and chromosomal rearrangements [17, 223]. These considerations underscore that therapeutic targeting of non-canonical nucleic acid structures will necessitate context-specific modulation rather than global inhibition, guided by robust predictive biomarkers. Progress in companion diagnostics will be essential: optimization and standardization of HRD detection assays are expected to improve identification of patients most likely to benefit from PARP inhibitors and DNA-damaging therapies, while emerging liquid biopsy approaches based on circulating tumor DNA hold promise for longitudinal monitoring of HRD status and therapeutic response, although the clinical utility of these strategies for this purpose remains to be established in prospective trials [262, 263, 269]. Furthermore, a deeper understanding of cell-type specificity in R-loops and D-loops regulation, particularly in post-mitotic neurons where both structures have been implicated in neurodegeneration and in the germline where D-loop dynamics are essential for meiotic fidelity, represents a priority area that is presently undercharacterized relative to its clinical relevance [129, 131].

Several priority research directions emerge from this comparative analysis. First, a high priority is the development of methods capable of simultaneously detecting R-loops and D-loops at the same genomic locus, ideally at single molecule resolution in living cells. Achieving this would transform our understanding of how these structures interact during transcription replication conflicts and at telomeres. Current approaches detect each structure in isolation using distinct biochemical enrichment strategies, precluding analysis of their co-occurrence and sequential dynamics at shared genomic sites. Second, systematic profiling of the post-translational modification landscape of shared regulatory factors (e.g., BRCA1, BRCA2, FANCM) under conditions that simultaneously perturb both R-loop and D-loop homeostasis would clarify whether these factors are coordinately regulated or independently allocated to each structure. Third, the application of live-cell imaging sensors including the RHINO system for R loops and catalytically inactive RAD51 reporters for D-loops within the same cellular system would enable real time visualization of R-loop to D-loop switching events, such as during the processing of transcription replication conflicts into HR intermediates [318]. Fourth, computational modeling integrating transcription rate, DNA supercoiling, replication timing, and recombinase kinetics could generate quantitative predictions of R-loop and D-loop co-occurrence that are currently beyond experimental resolution [198]. Finally, the clinical translation of these insights will require prospective studies to determine whether combined R-loop and D-loop biomarker panels (e.g., S9.6 immunostaining combined with RAD51 foci quantification) offer superior prognostic or predictive value compared to single-structure assays in stratifying patients for DNA damage–targeted therapies [270].

Ultimately, the comparative study of R-loops and D-loops illustrates a fundamental principle of genome biology: the utilization of nucleic acid topology as a regulatory parameter. By dynamically balancing the formation, stability, and resolution of these non-canonical structures, cells can coordinate transcription, repair, and replication, maintaining a precarious equilibrium that, when disrupted, lies at the heart of human disease.

## Data Availability

No datasets were generated or analysed during the current study.

## Abbreviations

AOA2: Ataxia with Oculomotor Apraxia type 2
ALT: Alternative Lengthening of Telomeres
BIR: Break-Induced Replication
BRCA1/2: Breast Cancer gene 1/2
BTR: BLM–TOP3α–RMI1/RMI2 complex
Cas9: CRISPR-associated protein 9
ccc-dsDNA: Covalently closed circular double-stranded DNA
CCCTC: CCCTC-binding factor
CDK: Cyclin-dependent kinase
ChIP: Chromatin immunoprecipitation
ChIP-seq: Chromatin Immunoprecipitation Sequencing
circRNA: Circular RNA
CSB1: Conserved Sequence Block 1
CTCF: CCCTC-binding factor
DDR: DNA Damage Response
DDX5: DEAD-box helicase 5
D-loop: Displacement loop
DRIP-seq: DNA: RNA Immunoprecipitation Sequencing
DSB: Double-Strand Break
eRNA: Enhancer RNA
FTD: Frontotemporal dementia
G4: G-quadruplex
HDR: Homology-Directed Repair
hDNA: Heteroduplex DNA
HSP: Heavy-Strand Promoter
HR: Homologous Recombination
HRD: Homologous Recombination Deficiency
IGF2BPs: Insulin-like growth factor 2 mRNA-binding proteins
i-loop: Internal loop
LSP: Light-Strand Promoter
lncRNA: Long non-coding RNA
LSD1: Lysine-specific demethylase 1 A
m6A: N6-methyladenosine
m5C: 5-methylcytosine
MC1R: Melanocortin 1 Receptor
METTL3: Methyltransferase-like 3
MRN complex: Mre11-Rad50-Nbs1 complex
mtDNA: Mitochondrial DNA
NCR: Non-Coding Region
NF-κB: Nuclear factor kappa B
NHEJ: Non-Homologous End Joining
OH: Origin of replication (Heavy strand)
POLγ: DNA polymerase gamma
RAD51/52/54: Radiation sensitive 51/52/54
RITOLS: Ribonucleotide incorporation throughout the lagging strand
R-loop: RNA: DNA hybrid loop
RNase H: Ribonuclease H
RPA: Replication Protein A
RTEL1: Regulator of telomere elongation helicase 1
SDSA: Synthesis-Dependent Strand Annealing
SETX: Senataxin
sgRNA: Single-guide RNA
SMN: Survival of motor neuron
SMX: SLX4–SLX1–MUS81–EME1–XPF–ERCC1 complex
ssDNA: Single-stranded DNA
STR: Short Tandem Repeat
TAS: Termination-Associated Sequence
TERRA: Telomeric repeat-containing RNA
T-loop: Telomere loop
TOP1: DNA topoisomerase I
TRC: Transcription–replication conflict
TRF1/2: Telomeric repeat-binding factor 1/2
TUG1: Taurine up-regulated gene 1
TWINKLE: Mitochondrial DNA helicase
YTHDC1: YTH domain-containing protein 1
YTHDF2: YTH N6-methyladenosine RNA binding protein 2
